# Chronologically modified androgen receptor in recurrent castration-resistant prostate cancer and its therapeutic targeting

**DOI:** 10.1126/scitranslmed.abg4132

**Published:** 2022-06-15

**Authors:** Mithila Sawant, Kiran Mahajan, Arun Renganathan, Cody Weimholt, Jingqin Luo, Vandna Kukshal, Joseph M. Jez, Myung Sik Jeon, Bo Zhang, Tiandao Li, Bin Fang, Yunting Luo, Nicholas J. Lawrence, Harshani R. Lawrence, Felix Y. Feng, Nupam P. Mahajan

**Affiliations:** 1 Department of Surgery, Washington University in St. Louis, St. Louis, MO 63110, USA.; 2 Division of Urologic Surgery, Washington University in St. Louis, St. Louis, MO 63110, USA.; 3 Siteman Cancer Center, Washington University in St. Louis, Cancer Research Building, 660 Euclid Ave., St. Louis, MO 63110, USA.; 4 Department of Anatomic and Clinical Pathology, Washington University in St. Louis, St. Louis, MO 63110, USA.; 5 Department of Biology, Washington University in St. Louis, One Brookings Drive, St. Louis, MO 63110, USA.; 6 Bioinformatics Research Core, Center of Regenerative Medicine, Department of Developmental Biology, Washington University in St. Louis, St. Louis, MO 63110, USA.; 7 Drug Discovery Department, Moffitt Cancer Center, Department of Oncologic Sciences, University of South Florida, 12902 USF Magnolia Drive, Tampa, FL 33612, USA.; 8 Helen Diller Family Cancer Research Building, 1450 Third Street, Room 383, University of California, San Francisco, CA 94158, USA.

## Abstract

Resistance to second-generation androgen receptor (AR) antagonists such as enzalutamide is an inevitable consequence in patients with castration-resistant prostate cancer (CRPC). There are no effective therapeutic options for this recurrent disease. The expression of truncated AR variant 7 (AR-V7) has been suggested to be one mechanism of resistance; however, its low frequency in patients with CRPC does not explain the almost universal acquisition of resistance. We noted that the ability of AR to translocate to nucleus in an enzalutamide-rich environment opens up the possibility of a posttranslational modification in AR that is refractory to enzalutamide binding. Chemical proteomics in enzalutamide-resistant CRPC cells revealed acetylation at Lys^609^ in the zinc finger DNA binding domain of AR (acK609-AR) that not only allowed AR translocation but also galvanized a distinct global transcription program, conferring enzalutamide insensitivity. Mechanistically, acK609-AR was recruited to the *AR* and *ACK1/TNK2* enhancers, up-regulating their transcription. ACK1 kinase–mediated AR Y267 phosphorylation was a prerequisite for AR K609 acetylation, which spawned positive feedback loops at both the transcriptional and posttranslational level that regenerated and sustained high AR and ACK1 expression. Consistent with these findings, oral and subcutaneous treatment with ACK1 small-molecule inhibitor, (*R*)-9b, not only curbed AR Y267 phosphorylation and subsequent K609 acetylation but also compromised enzalutamide-resistant CRPC xenograft tumor growth in mice. Overall, these data uncover chronological modification events in AR that allows prostate cancer to evolve through progressive stages to reach the resilient recurrent CRPC stage, opening up a therapeutic vulnerability.

## INTRODUCTION

Therapies directed against the androgen receptor (AR) signaling axis are the mainstay of treatment regime for patients with early- or later-stage metastatic prostate cancer (1). Often, the first line of treatment for patients with localized disease is androgen deprivation therapy (ADT). Although ADT reduces the tumor burden and prolongs patient survival, it is only transiently effective. Almost all patients recur and exhibit a metastatic disease, castration-resistant prostate cancer (CRPC). CRPC underlines the pitfall of AR functional targeting by ADT, which is compensated via refueling- enhanced AR signaling. AR mutations, gene/enhancer amplification, modifications such as Tyr phosphorylation, coactivator overexpression, and intratumoral de novo androgen synthesis are among the well-studied schemes that occur in CRPC tumors (2–9). With such an obligate dependence on AR, next-generation AR antagonists such as enzalutamide, which prevents AR nuclear translocation (10), and abiraterone, which compromises androgen synthesis (11), have received widespread acceptance as CRPC remedies. Unfortunately, these therapies share a similar fate: Patients who respond to enzalutamide and abiraterone acquire resistance in a relatively short time. One suggested mechanism of such resistance is increased expression of truncated AR variant 7 (AR-V7), with an N-terminal transactivation domain and a DNA binding domain but lacking a ligand-binding domain (12–14). The frequency of AR-V7 expression in metastatic CRPC (mCRPC) is about 18.3% (15), suggesting the existence of an additional mechanism involved in resistance to AR antagonists. Recently, amplification in the AR enhancer region located 624 kb upstream of the start site was reported in enzalutamide-, abiraterone-, and apalutamide-treated CRPC tumors (16–18). In addition, an epigenetic modification of the *AR* enhancer has also been reported wherein histone Y88 phosphorylation marks were deposited 94, 114, and 132 kb upstream of the start site, causing increased AR and AR-V7 expression in CRPC (19). Although continuous reliance on enhanced AR signaling has emerged as the predominant modus operandi for drug-resistant CRPC, the precise mechanism by which AR (or AR-V7) up-regulation translates into overcoming sensitivity to AR antagonists is not completely understood.

Here, we report a modification in AR K609 acetylation that depends on AR Y267 phosphorylation exerted by nonreceptor tyrosine kinase Activated Cdc42-associated kinase 1 (ACK1). This “dual- modified AR” regulates the expression of AR and ACK1 that phosphorylates AR at Y267, thereby creating a self-sustaining circuit that maintains high expression of AR and ACK1. In this study, we sought to unravel the mechanistic details of successive interdependent AR modification events that impart resistance to AR antagonists and further explored whether targeting the ACK1-AR nexus with small-molecule inhibitor (*R*)-**9b** would escalate the antineoplastic response in recurrent CRPC.

## RESULTS

### Identification of AR acetylation at K609 in enzalutamide-resistant CRPCs

**Fig. 1.**
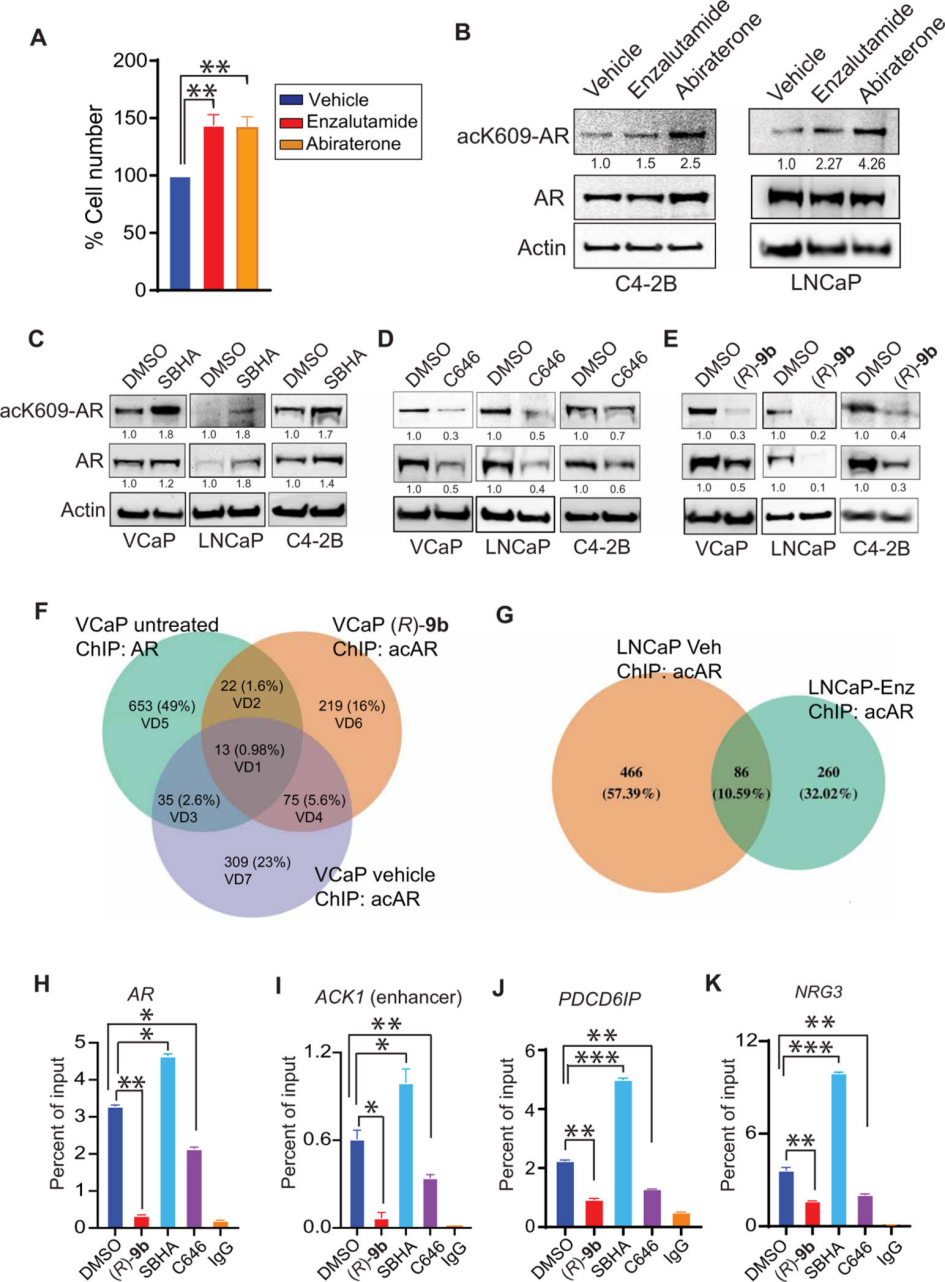
AR Lys^609^ acetylation is enhanced in enzalutamide- and abiraterone-resistant CRPC. (**A**) Resistant C4–2B cells were treated with 7 μM enzalutamide or abiraterone for 7 days, and the cell viability was measured via a trypan blue exclusion assay (*n* = 3, three replicates). (**B**) Enzalutamide-resistant C4–2B and LNCaP cells were subjected to IP using acK609-AR antibody, followed by immunoblotting with AR antibodies (top). The same lysates were also subjected to immunoblotting with AR and actin antibodies (bottom). (**C** to **E**) Modulation of acK609-AR with (C) SBHA (1 μM, 24 hours), (D) C646 (5 μM, 24 hours), and (E) ACK1 inhibitor (*R*)-**9b** (5 μM, 24 hours) treatment in VCaP, LNCaP, and C4–2B prostate cancer cells. Endogenous acK609-AR was detected by IP using ac609-AR antibody, followed by immunoblotting with AR antibodies (top). Relative expression of acK609-AR and AR is shown. For (B to E), shown below each blot is the densitometric measurement of change in abundance relative to control. (**F**) Venn diagrams (VDs) summarizing the overlap between sites bound by acK609-AR and AR in VCaP cells. (**G**) VDs summarizing the overlap between sites bound by acK609-AR in enzalutamide- and vehicle-treated LNCaP cells. (**H** to **K**) VCaP cells were treated overnight with vehicle, (*R*)-**9b** (5 μM), SBHA (1 μM), or C646 (5 μM), and chromatin was immunoprecipitated (ChIP) with ac609-AR antibody, followed by qPCR with primers corresponding to (H) *AR* enhancer AREM1, (I) *ACK1* enhancer, (J) *PDCD6IP* intron, or (K) *NRG3* intron. For (H to K), *n* = 2, three replicates; representative data are shown. Data are represented as means ± SEM. *P < 0.05, **P < 0.01, and ***P < 0.001, Student’s *t* test. DMSO, dimethyl sulfoxide; IgG, immunoglobulin G.

To investigate molecular mechanisms of CRPC drug resistance, we established enzalutamide- and abiraterone-resistant C4–2B cells, a CRPC cell line. In addition to being drug insensitive, the resistant CRPC cells exhibited marked growth in the presence of high concentrations of these two widely used AR antagonists (Fig. 1A). We performed an unbiased chemical proteomic analysis to determine whether a posttranslationally modified AR promotes differential association with enzalutamide. We attached a tether suitable for securing enzalutamide, and the methyl group of enzalutamide was replaced with a polyethylene glycol linker with a terminal amine attached to biotin (biotin-X-CO_2_H), allowing bound proteins to be captured by streptavidin- coated Sepharose beads (figs. S1 and S2). The enzalutamide–biotin conjugate– bound streptavidin Sepharose beads were incubated with nuclear lysates prepared from enzalutamide-resistant C4–2B cells. Beads linked to another inhibitor, (*R*)-**9b**, were used as a negative control (figs. S1 and S2). The bound proteins were eluted and subjected to mass spectrometry. This drug affinity chromatography approach enabled by downstream mass spectrometry (liquid chromatography–tandem mass spectrometry) led to the identification of AR that was acetylated at K609 (fig. S1).

K609 in the second zinc finger of the DNA binding domain (DBD) (fig. S3A) is conserved in AR of various mammals (fig. S3B, bottom). K609 is also conserved in the estrogen receptor (ER), glucocorticoid receptor (GR), and progesterone receptor (PR) (fig. S3B, top). To determine the physiological relevance of AR acetylation at K609 (acK609-AR), we developed monoclonal antibodies recognizing this modification. The acK609-AR antibodies recognized acetylated AR peptides but failed to bind corresponding unmodified AR peptide (fig. S3C). The antibody displayed excellent specificity for acK609-AR compared to unmodified AR and to other acetylated peptides derived from adenosine triphosphate synthase, HOXB13, and histone H3 (fig. S3C). A competitive binding assay was also performed wherein acK609-AR antibody was incubated with three distinct acetyl antibodies, acHOXB13, acH3K130, and acK609-AR, followed by incubation with membranes spotted with acK609-AR peptide. acHOXB13 and acH3K130 antibodies failed to compete for acK609-AR antibody binding to the corresponding peptide, reiterating the recognition specificity of the acK609-AR antibody (fig. S3D). The specificity of the antibody was further validated by generating sections from fixed LAPC4, VCaP, LNCaP, and C4–2B cells treated with either vehicle or (*R*)-**9b**, which inhibits AR K609 acetylation, followed by immunohistochemical (IHC) staining using acK609-AR antibody. As displayed in fig. S3E, vehicle-treated cells exhibited strong intensity of acK609-AR staining in comparison to (*R*)-**9b**–treated cells. Cells stained with just primary or secondary antibody were used as controls to negate background staining (fig. S3F). The expression of acK609-AR compared to total AR expression in LNCaP, VCaP, and C4–2B cell lines in the presence of enzalutamide was found to be 0.1, 0.08, and 0.12%, respectively (fig. S3G), which increased to 0.27% upon histone deacetylase 3 (HDAC3) inhibition in C4–2B cells (fig. S3H). The acetylation of AR is consistent with global stoichiometry of acetylated proteins (20).

To examine whether AR K609 acetylation directly correlated with AR antagonist treatment, we treated C4–2B and LNCaP cells with enzalutamide and abiraterone, which revealed increased acK609-AR (Fig. 1B). We examined the effect of the HDAC1 and HDAC3 inhibitor suberoyl bis-hydroxamic acid (SBHA), HDAC3 inhibitor RGFP966, and the p300 histone acetyltransferase (HAT) inhibitor C646. Prostate cancer cell lines VCaP, LNCaP, and C4–2B were treated with SBHA and VCaP; LNCaP was treated with RGFP966, resulting in increased acK609-AR upon HDAC1/3 inhibition (Fig. 1C and fig. S4B). In contrast, down- regulation in acK609-AR was seen upon p300 HAT inhibition (Fig. 1D), suggesting a dynamic regulation of this modification by acetyl transferase and deacetylase enzymes in prostate cancer cells. Furthermore, silencing of mRNAs encoding HDACs 1, 2, and 3, as well as p300 and CREB-binding protein (CBP), was performed by transfecting small interfering RNAs into VCaP and LNCaP cells to delineate the responsible deacetylase and acetyltransferase. acK609-AR buildup was observed after silencing of HDAC1 and HDAC3, whereas silencing of both CBP and p300 complex diminished acK609-AR (fig. S4C). The silencing of mRNAs encoding HDACs 1, 2, 3, p300, and CBP was confirmed by quantitative reverse transcription polymerase (Pol) chain reaction (qRT-PCR) (fig. S4D). Together, these data indicate that p300/CBP acetylates AR, whereas HDAC1 and HDAC3 may be causing its deacetylation.

Our previous studies demonstrated a role of ACK1 kinase in multiple malignancies, including prostate cancer (21–26). ACK1 is activated (Y284-phosphorylated) in prostate cancers, with highest expression in CRPCs (9, 27). Seven in absentia homolog (SIAH) ubiquitin ligases target ACK1 for proteasomal degradation (28). Activated ACK1 epigenetically modifies the *AR* gene locus by depositing histone H4 Tyr^88^ marks in the *AR* enhancer, a crucial step for efficient transcription of AR and AR-V7 in CRPCs (19). This finding stimulated the development of an ACK1 small-molecule inhibitor, (*R*)-**9b**, which exhibits both good potency (median inhibitory concentration, 47 nM) and selectivity (19). ACK1 interacts with heat shock protein 90 α/β (29, 30), which is also known to bind to AR (31), thus bringing ACK1 in close proximity of AR. Because ACK1 phosphorylates AR in response to androgen deprivation, which promotes CRPC (7, 9, 32), we sought to determine whether the subsequent post-CRPC modification of AR was also influenced by ACK1. To assess the impact of (*R*)-**9b** on the abundance of acK609-AR, we subjected VCaP, LNCaP, and C4–2B cells to (*R*)-**9b** treatment. A decrease in acK609-AR was observed upon (*R*)-**9b** treatment (Fig. 1E). Total AR exhibited a direct correlation with acK609-AR (Fig. 1E, second panel), suggesting that ACK1 may regulate acK609-AR, which may play a role in maintaining AR. In addition, (*R*)-**9b** exhibited minimal effect on the total global acetylation in prostate cancer cells (fig. S4A), suggesting its major role in disrupting ACK1 mediated AR regulation.

### acK609-AR regulates AR and ACK1 expression in enzalutamide-resistant CRPCs

To examine the transcription program regulated by acK609-AR protein, chromatin prepared from vehicle- or (*R*)-**9b**–treated enzalutamide-resistant VCaP cells was immunoprecipitated (ChIP) with acK609-AR antibody, followed by next-generation sequencing (ChIP-seq). Peak analysis revealed acK609-AR binding to 727 distinct sites; acK609-AR peaks were primarily annotated in intergenic regions, including enhancers and introns (fig. S5A and table S1). The genes that exhibit acK609-AR binding, as well as those that were regulated upon (*R*)-**9b** treatment, are shown in data file S1. For comparison, we mined AR ChIP-seq data for VCaP cells (GSE28950, GSM717391, and GSM717392) and identified the genes that exhibit AR binding in these cells (data file S1). Furthermore, Venn diagram (VD) analysis revealed an occupancy of acK609-AR at 309 unique sites (VD7), with 88 sites (VD1 and VD4) shared between vehicle- and (*R*)-**9b**–treated cells (Fig. 1F and data file S2). Only 48 sites (VD1 and VD3) overlapped between acK609-AR and AR binding, and the large majority of AR peaks (677 peaks) did not overlap with acK609-AR peaks (Fig. 1F and data file S2), Furthermore, we cultured enzalutamide-sensitive LNCaP cells in the presence of the drug to generate an isogenic enzalutamide-resistant cell line (Fig. 1B). ChIP-seq revealed that similar to enzalutamide-resistant VCaP cells, acK609-AR initiated a transcription program in enzalutamide-resistant LNCaP cells (Fig. 1G, table S1, and data file S3). The overlap in the VCaP and LNCaP acK609-AR binding sites is shown in fig. S5B.

The acK609-AR binding motifs predicted by HOMER, including the corresponding relative score, sequence, and transcription factors, are shown in figs. S5C to S8 and show a distinct set of motifs used by acK609-AR. When the acK609-AR motifs in enzalutamide-resistant VCaP (fig. S5C) and enzalutamide-resistant LNCaP cells (fig. S7) were compared, it was observed that 7 of 12 motifs in VCaP cells were shared with LNCaP cells [ZNF189, zinc finger protein X-linked (ZFX), TEA domain transcription factor 3 (TEAD3), SOX9 (SRY-box transcription factor 9), signal transducers and activators of transcription 6 (STAT6), beta-glucuronidase-like protein (SMA3), and forkhead box protein H1 (FOXH1)], indicating that multiple acK609-AR recognition sequences may be common between these two resistant cell lines.

The enzalutamide-resistant VCaP ChIP-seq data indicate that *HSD17B11*, *SIM2*, *ZRANB3*, and *ADAMTSL3* are among the top genes with acK609-AR binding (data file S1 and fig. S9, A to D). The entire AR locus seemed to be recognized by acK609-AR, which showed a marked decrease upon (*R*)-**9b** treatment (fig. S9E). acK609-AR binding was also observed in the *ACK1/TNK2* gene enhancer (fig. S9G). In addition, acK609-AR binding at the *AR* enhancer was observed for the AREM1 site, which we previously reported as the pY88-H4 deposition site (fig. S9F) (19). Other prominent sites of acK609-AR binding included the first introns of the *NRG3* and the *PDCD6IP* genes [fig. S9 (H and I, respectively)].

We assessed the molecular advantage imparted by AR K609 acetylation with a ChIP-qPCR assay using acK609-AR antibodies. ChIP-qPCR confirmed acK609-AR binding at *AR* and *ACK1* enhancers (Fig. 1, H and I) and at the introns of *PDCD6IP* and *NRG3* (Fig. 1, J and K), which was abrogated by (*R*)-**9b** treatment. This binding was enhanced by the HDAC inhibitor SBHA, which prevented AR K609 deacetylation, and was diminished by the HAT inhibitor E (Fig. 1, H to K). These data further confirm that AR acetylation at K609 is crucial for recruitment at these sites.

To determine whether enzalutamide- and abiraterone-resistant C4–2B cells displayed differential binding of acK6090-AR, ChIP-qPCR was performed. Both drugs induced recruitment of acK609-AR onto *AR*, *ACK1*, *KLK3* (PSA), and *NRG3* genomic loci (fig. S10, A to D). These results indicate that enzalutamide- and abiraterone-resistant CRPCs introduce posttranslational alterations in the DBD of AR, facilitating its enhanced recruitment to a distinct set of genetic loci.

### K609 acetylation is required for AR recruitment to target genes

**Fig. 2.**
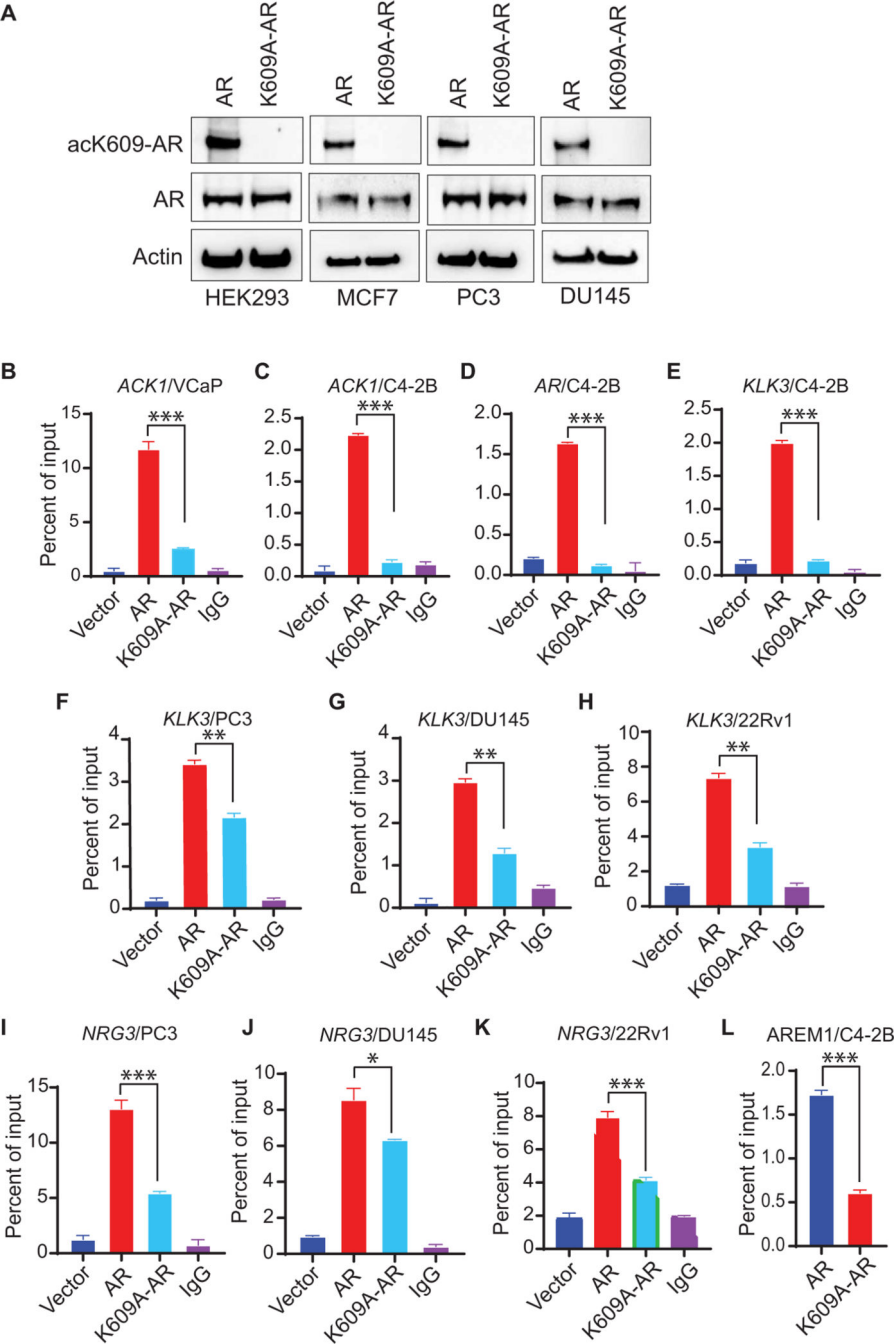
K609 acetylation–deficient AR exhibits poor binding to responsive genes. (**A**) HEK293, MCF7, PC3, and DU145 cells were transfected with FLAG-tagged AR or K609A-AR mutant. Lysates were subjected to IP with acK609-AR antibodies, followed by immunoblotting with AR antibodies. (**B** to **E**) VCaP and C4–2B cells were infected with retroviral plasmids for vector, AR, or K609A-AR and subjected to ChIP using FLAG beads, followed by qPCR using primers corresponding to *ACK1* (B and C), *AR* (D), or *KLK3* (E) enhancers. (**F** to **K**) PC3, DU154, and 22Rv1 cells were transfected with vector-, AR-, or K609A-AR–expressing plasmids and subjected to ChIP using FLAG beads, followed by qPCR using primers corresponding to the *KLK3* enhancer (F to H) or *NRG3* intron (I to K). (**L**) Retrovirally infected C4–2B cells were subjected to ChIP using RNA Pol II antibody, followed by qPCR using AREM1 primers. For (B to L), *n* = 2, three replicates; representative data are shown. Data are represented as means ± SEM. *P < 0.05, **P < 0.01, and ***P < 0.001, Student’s *t* test.

To determine the mechanistic role of K609 acetylation, we generated constructs expressing FLAG-tagged AR and a K609A (lysine-to- alanine substitution at site 609) mutant of AR (K609A-AR) and transfected the constructs in prostate (PC3 and DU145), breast (MCF7), and human embryonic kidney (HEK) 293 cells with low or undetectable AR expression. The K609A-AR constructs exhibited decrease in AR K609 acetylation (Fig. 2A). We next examined the recruitment of AR K609 acetylation at *ACK1*, *AR*, *PSA*, and *NRG3* genetic loci by retroviral infection of enzalutamide-resistant C4–2B and VCaP cell lines with the FLAG-tagged constructs. After transfection, cells were grown in charcoal-stripped fetal bovine serum media (androgen deprivation, followed by ChIP-qPCR). Compared with AR, the acetylation-deficient K609A-AR mutant exhibited decreased binding at *ACK1* (Fig. 2, B and C), *AR* (Fig. 2D), *KLK3* (Fig. 2, E to H), and *NRG3* (Fig. 2, I to K) sites. Furthermore, decreased RNA Pol II binding to the AR enhancer AREM1 was seen in the K609A mutant AR-expressing cells (Fig. 2L). As an additional control, we generated a K609Q (lysine-to-glutamine substitution) mutant of AR that mimics the acetylation. The K609Q-AR mutant strengthened AR coprecipitation with ACK1 protein (fig. S11A), and the acetylation-mimicking K609Q-AR mutant exhibited increased binding to AR target gene KLK3/*PSA* enhancer (fig. S11, B and C).

**Fig. 3.**
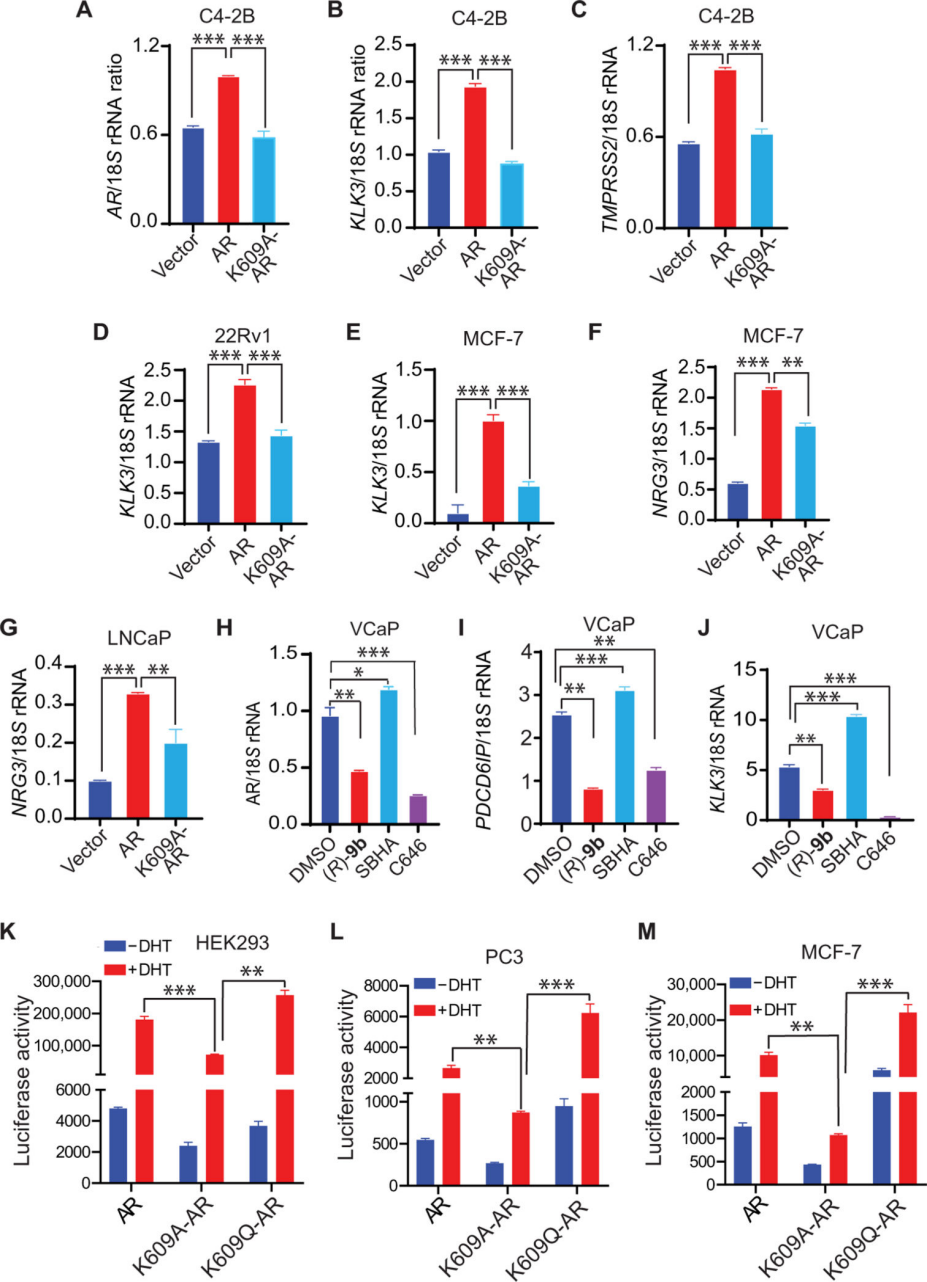
AR acetylation at K609 up-regulates target gene transcription. (**A** to **C**) C4–2B cells were retrovirally infected with vector, AR, or K609A-AR constructs. After 96 hours, RNA was extracted, followed by qRT-PCR using primers corresponding to *AR* (A), *KLK3* (B), or *TMPRSS2* (C). (**D** to **G**) 22Rv1, MCF7, and LNCaP cells were transfected with vector, AR, and K609A-AR constructs. Forty-eight hours after transfection, RNA was isolated, followed by qRT-PCR using primers corresponding to PSA [(D) 22Rv1; (E) MCF7] or NRG3 [(F) MCF7; (G) LNCaP]. (**H** to **J**) VCaP cells were treated overnight with (*R*)-**9b** (5 μM), SBHA (1 μM), or C646 (5 μM). RNA was isolated, followed by qRT-PCR using primers corresponding to AR (H), *PDCD6IP* (I), and *KLK3* (J). (**K** to **M**) HEK293 (K), PC3 (L), and MCF7 (M) cells were transfected with AR, K609A-AR mutant, or K609Q-AR mutant and the ARR2PB-luciferase reporter construct. Cells were treated with or without 10 nM DHT overnight in serum-free media, and luciferase activity was determined 48 hours after transfection. For (A to M), *n* = 2, three replicates; representative data are shown. Data are represented as means ± SEM. *P < 0.05, **P < 0.01, and ***P < 0.001, Student’s *t* test.

To examine the transcriptional outcome of K609-acetylated AR in an androgen-deficient environment, its downstream targets were explored. *AR*, *KLK3*, *TMPRSS2*, and *NRG3* transcripts were notably diminished in cells expressing the K609A AR mutant (Fig. 3, A to G). To determine the influence of this modification in an endogenous context, serum-starved VCaP cells were treated with (*R*)-**9b**, SBHA, or C646. Both (*R*)-**9b** and C646 exerted an inhibitory effect on the AR (Fig. 3H), *PDCD6IP* (Fig. 3I), and *KLK3* (Fig. 3J), whereas SBHA caused a spike in these transcripts (Fig. 3, H to J). In addition, (*R*)-**9b** caused down-regulation of the acK609-AR–regulated genes *HSD17B11*, *SIM2*, *ZRANB3*, and *ADAMTSL3* (fig. S11, D to G). Together, these data demonstrate that AR K609 acetylation in the DBD is critical for efficient recruitment and transcriptional activation of a distinct set of genetic loci, including AR itself. acK609-AR promotes the expression of crucial CRPC regulators such as AR and ACK1 in an enzalutamide- or abiraterone-rich environment, creating a regenerative scheme to sustain their expression even in the presence of AR antagonists.

To investigate the contribution of Lys^609^ acetylation during AR transcription activity in the presence of its ligand dihydrotestosterone (DHT), AR-deficient HEK293, PC3, and MCF7 cells were cotransfected with AR, K609A-AR, or K609Q-AR mutants and an ARR2PB-luciferase reporter construct. DHT treatment caused an increase in the luciferase activity of AR; however, luciferase activity of the acetylation-deficient K609A-AR mutant was compromised (Fig. 3, K to M, and fig. S12, A and B). In contrast, the K609Q acetylation–mimicking mutant exhibited a significant increase in luciferase activity compared to the K609A-AR mutant (*P* < 0.01 and *P* < 0.001; Fig. 3, K to M).

To negate the effect of endogenous AR protein, VCaP and C4–2B cells were transfected with AR 5′ untranslated region–specific silencing oligos, followed by retroviral infection with an AR- or K609A mutant–expressing construct (fig. S12C). Enhanced resistance was exhibited by the AR-expressing cells upon enzalutamide treatment, whereas K609A mutant AR-expressing cells showed reduced proliferation (fig. S12D). In addition, we depleted endogenous AR, introduced K609A and K609Q AR mutants as described above, and then transfected the cells with an ARR2PB-Luc reporter construct. A luciferase assay revealed decreased AR activity in K609A-expressing cells, whereas K609Q mutant–expressing cells exhibited increased AR activity (fig. S12E). Overall, the inability of the K609 acetylation–deficient mutant to optimally trigger AR transcription activity even in the presence of DHT signifies the critical importance of K609 acetylation not only in an androgen-deficient environment but also in mounting a response to androgen.

### ACK1-mediated AR Tyr phosphorylation is a prerequisite for AR K609 acetylation

**Fig. 4.**
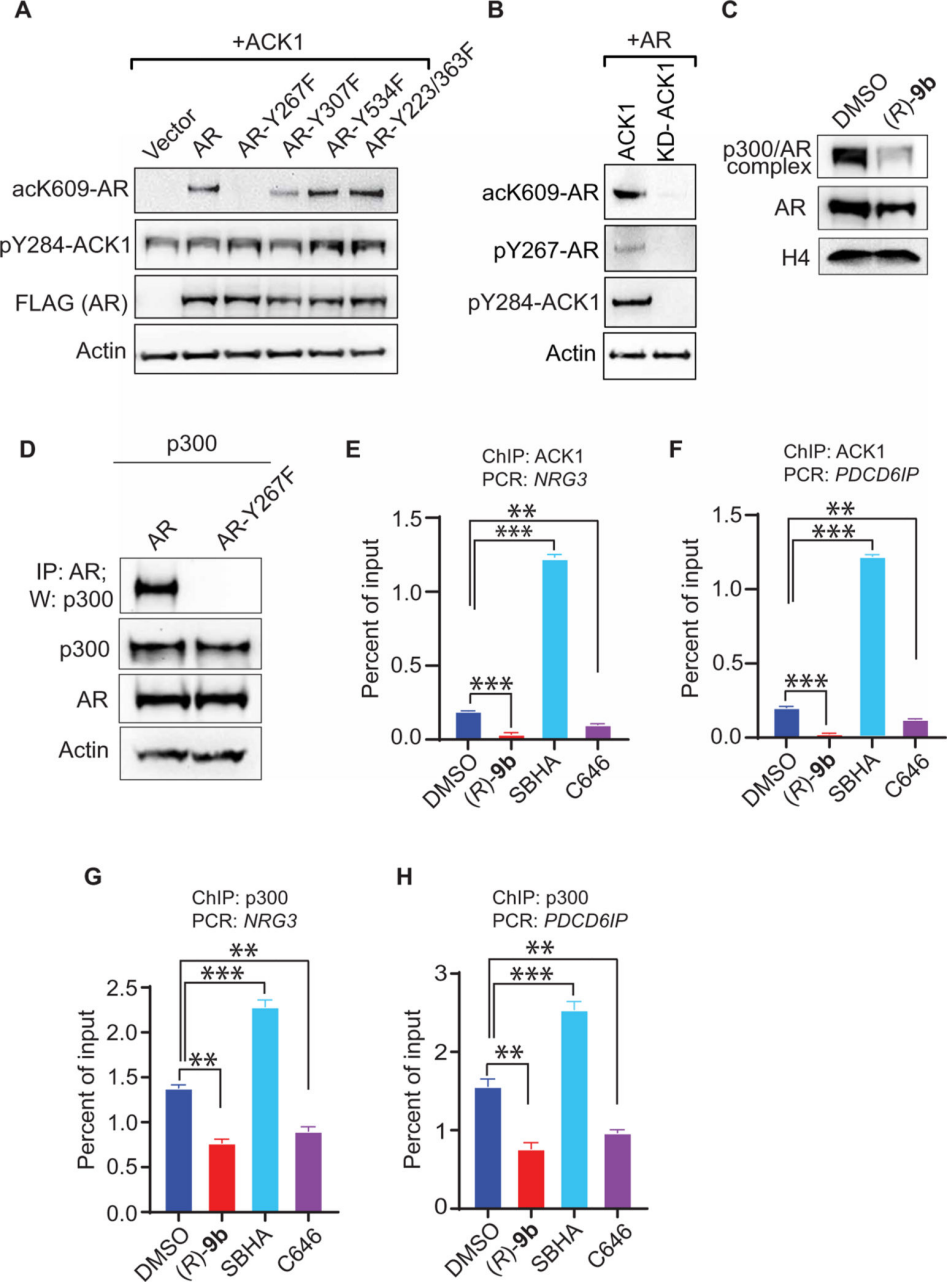
AR Tyr phosphorylation by ACK1 is required for acetylation at K609. (**A**) HEK293 cells were cotransfected with vector, ACK1 [hemagglutinin (HA) tagged], FLAG-tagged AR, Y267F-AR, Y307F-AR, Y534F-AR, or Y223/363F-AR mutants. After 48 hours, cell lysates were subjected to IP using acK609-AR antibody, followed by immunoblotting with FLAG antibody (top). The same lysates were subjected to immunoblotting with pY284-ACK1, FLAG, and actin antibodies (bottom). (**B**) HEK293 cells were transfected with FLAG-tagged AR and ACK1 or KD-ACK1 plasmid. After 48 hours of transfection, lysates were subjected to IP using acK609-AR antibody, followed by immunoblotting with FLAG antibody (top). (**C**) VCaP cells were treated with vehicle (DMSO) or (*R*)-**9b**, and lysates from nuclear fractionations were subjected to IP using p300 antibody, followed by immunoblotting with AR antibody (top). (**D**) HEK293 cells were cotransfected with p300 plasmid and AR or AR-Y267F mutant, and the lysates were subjected to IP with AR antibody and immunoblotting with p300 antibody. (**E** to **H**) VCaP cells were treated with vehicle, (*R*)-**9b**, SBHA, or C646, and the lysates were subjected to ChIP using ACK1 (E and F) or p300 antibodies (G and H), followed by qPCR with primers for *NRG3* (E and G) or *PDCD6IP* (F and H) introns. For (E to H), *n* = 2, three replicates; representative data are shown. Data are represented as means ± SEM. *P < 0.05, **P < 0.01, and ***P < 0.001, Student’s *t* test.

In a model of human AR bound to androgen direct-repeat response element (ARE), the K609 from each monomer is positioned within the minor groove to interact with the phosphate backbone of the response element (fig. S13, A to C). It has been reported that AR is phosphorylated at multiple sites, including Y267, Y307, Y363, and Y534 (7, 8). To obtain mechanistic insight into a potential regulatory role of AR Tyr phosphorylation in AR-K609 acetylation, ACK1 was coexpressed with AR and its phosphorylation mutants. Increased AR K609 acetylation was observed upon ACK1 coexpression; however, the Y267F mutant of AR exhibited a complete loss of AR K609 acetylation (Fig. 4A). In contrast, AR mutants Y307F, Y534F, and Y223F/Y363F exhibited little to no change in AR K609 acetylation, indicating that AR Y267 phosphorylation precedes K609 acetylation. We verified the dependence of AR K609 acetylation on Y267 phosphorylation by transfecting HEK293 cells with an FLAG-tagged AR-expressing construct in the presence of ACK1 or kinase-dead ACK1 plasmid (KD-ACK1). Immunoprecipitation (IP) with acK609-AR antibody followed by immunoblotting with FLAG antibody revealed that the failure of KD-ACK to phosphorylate AR resulted in a lack of K609 acetylation of AR (Fig. 4B).

To obtain further mechanistic insights into the successive nature of AR modifications, we investigated whether phosphorylation of AR is needed for its recognition by HAT p300 to promote subsequent acetylation. VCaP cells were treated with (*R*)-**9b**, and IP was performed with p300 antibody, followed by immunoblotting with AR. An AR/p300 complex was observed in vehicle-treated samples; however, complex formation was compromised upon (*R*)-**9b** treatment (Fig. 4C). In addition, a co-IP of p300 with either AR or AR-Y267F revealed the ability of p300 to bind to AR, whereas AR-Y267F mutant failed to form a complex, reiterating that phosphorylation of AR at Y267 is a prerequisite for its acetylation and subsequent translocation into the nucleus, initiating downstream transcription activation (Fig. 4D). We further explored the ACK1/pAR/p300 association by detecting the assembly of this complex at target genes via ChIP with ACK1 and p300, followed by qPCR using *NRG3* and *PDCD6IP* loci-specific primers. We detected a pronounced binding of ACK1 and p300 at these sites, which was down-regulated upon treatment with the ACK1 inhibitor (*R*)-**9b** and p300 inhibitor C646. In contrast, ACK1 and p300 binding was up-regulated upon treatment with the HDAC1/3 inhibitor SBHA (Fig. 4, E to H). Together, these data indicate that the pAR/ACK complex recruits p300 to acetylate AR at K609, facilitating the binding of dual-modified AR at AR target genes to promote a distinct transcription program.

### Impeding the pACK1/acK609-AR axis represses enzalutamide/abiraterone-resistant CRPC tumor growth

**Fig. 5.**
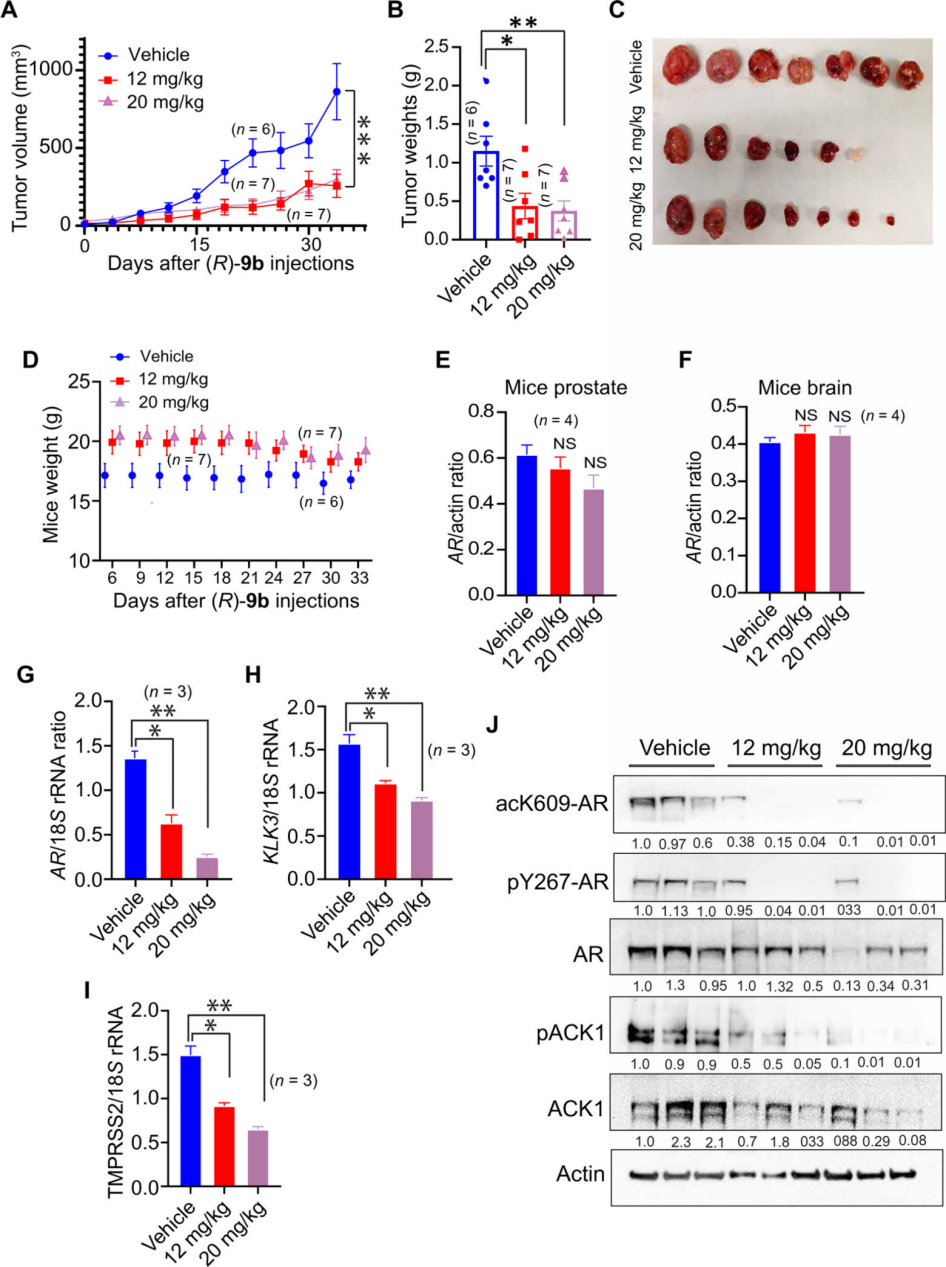
(*R*)-9b inhibits enzalutamide-resistant CRPC xenograft tumor growth in vivo. (**A**) Enzalutamide-resistant C4–2B cells were injected subcutaneously in castrated male SCID mice. Once tumors were palpable, he mice were treated subcutaneously with either vehicle (Captisol; *n* = 6) or (*R*)-**9b** at 12 (*n* = 7) or 20 mg/kg (*n* = 7) five times a week. Tumor volumes were measured twice a week. (B and C) Once they reached ~1000 mm^3^ in volume, the tumors were harvested and photographed, and their weights were recorded. Tumors 4 and 5 are from the same vehicle-treated mice. (**D**) Weights of vehicle- and (*R*)-**9b**–treated mice were recorded. (**E** and **F**) Prostates (E) and brains (F) of the mice were harvested, and RNA was prepared, followed by qRT-PCR (*n* = 4 each). (**G** to **I**) Tumors were harvested, and RNA was prepared, followed by qRT-PCR of *AR* (G), *KLK3* (H), and *TMPRSS2* (I) mRNAs (*n* = 3 mice each). Data are represented as means ± SEM. *P < 0.05, **P < 0.01, and ***P < 0.001. NS, not significant, Student’s *t* test. (**J**) Tumor lysates (*n* = 3) were IP using acK609-AR or pY267-AR antibodies, followed by immunoblotting by AR antibody (top two panels). pACK1, total AR, ACK1, and actin in the tumor lysates are shown in the bottom panels. Shown below each blot is the densitometric measurement of change in abundance relative to control.

To investigate the effect of inhibition of AR K609 acetylation on CRPC xenograft tumor growth, enzalutamide/abiraterone-resistant C4–2B cells were implanted subcutaneously in castrated male severe combined immunodeficient (SCID) mice. When the tumors became palpable (about 100 mm^3^ in size), the mice were randomized and injected with either vehicle [6% Captisol in phosphate-buffered saline (PBS)] or (*R*)-**9b** (12 or 20 mg/kg of body weight) five times a week. Tumor measurements were performed twice a week. CRPC tumor growth was compromised upon (*R*)-**9b** treatment, and the dose at 12 mg/kg was found to be sufficient to overcome CRPC tumor growth (Fig. 5A). On completion of the time points, the mice were humanely euthanized, and the tumors were harvested and weighed. (*R*)-**9b**–treated CRPC tumors displayed a reduction in weight, in contrast to the robust tumor growth seen in vehicle-treated mice (Fig. 5B). Excised tumors are shown in Fig. 5C. No change in body weight was observed in (*R*)-**9b**–treated mice (Fig. 5D), suggesting that (*R*)-**9b** is not toxic.

To further gauge the effect of (*R*)-**9b** on the host, total RNA was isolated from the prostate and brain of these mice. The brain was chosen because it is known to have AR expression, and it is important to determine whether (*R*)-**9b** passes the blood-brain barrier. No marked deviation from normal was observed in prostate or brain AR expression upon (*R*)-**9b** treatment (Fig. 5, E and F). In contrast, the prostate xenograft tumors exhibited a marked reduction in *AR*, *KLK3*, and *TMPRSS2* mRNAs compared with vehicle-injected mice (Fig. 5, G to I). The CRPC xenograft tumor lysates were subjected to immunoblotting, which demonstrated that (*R*)-**9b** treatment caused a marked decrease in pACK1 and ACK1 protein in comparison to the vehicle. This decline was accompanied by a corresponding suppression of pY267-AR and acK609-AR expression (Fig. 5J). Collectively, these data indicate that (*R*)-**9b** has no adverse side effects on the physiological well-being of the mice, as reflected by a lack of change in mice weight or AR abundance in host organs such as the prostate and brain; however, AR expression in CRPC tumors was specifically compromised by (*R*)-**9b**. Combined with our previously published findings that show specific activation of ACK1 in prostate tumor cells (7, 27), these data demonstrate that methodical ACK1 squelching by (*R*)-**9b** leads to a loss of acK609-AR, resulting in a comprehensive response against CRPC.

### Oral administration of (*R*)-9b is effective in suppressing prostate cancer growth

**Fig. 6.**
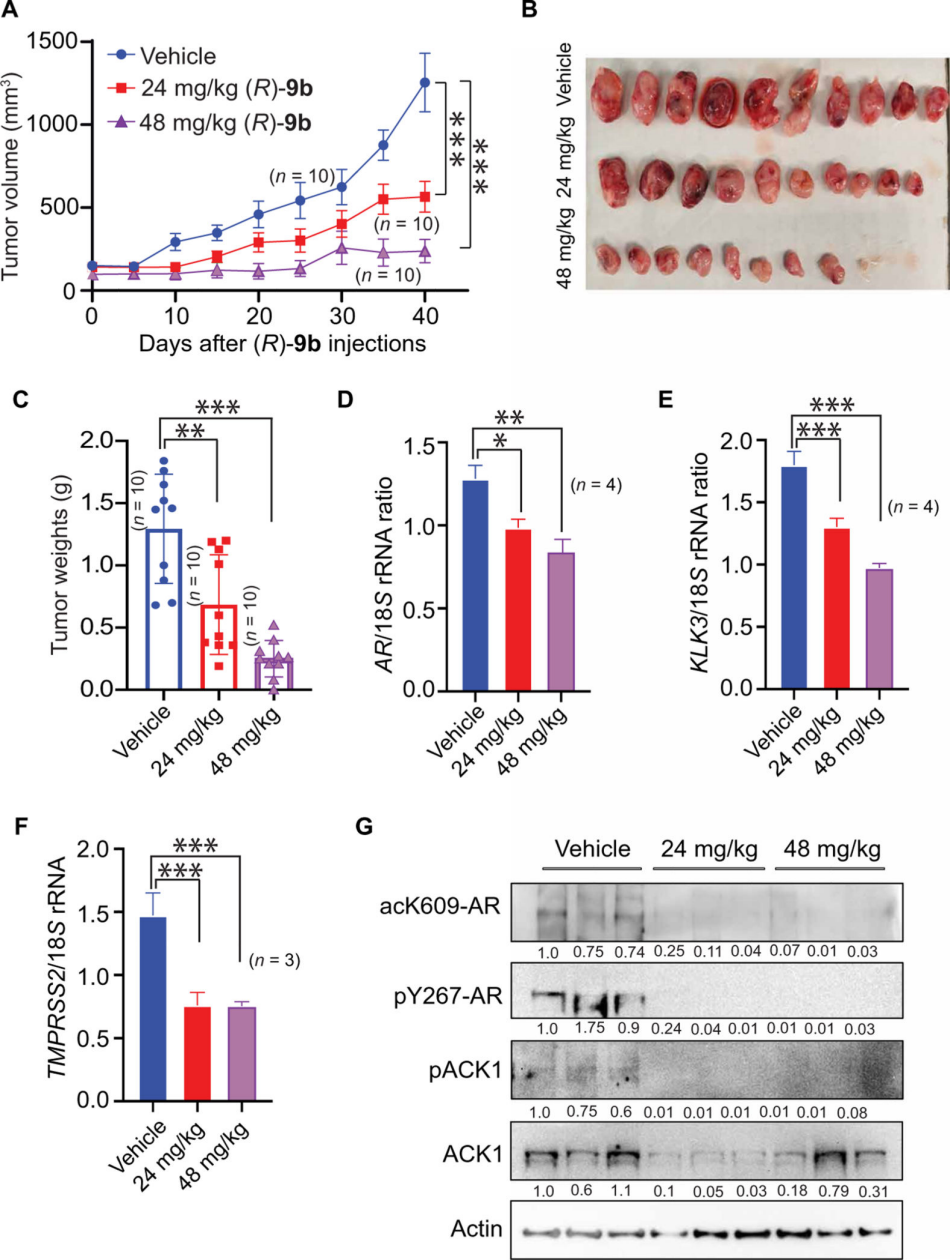
Oral bioavailability of (*R*)-9b in suppressing enzalutamide-resistant xenograft tumor growth. (**A**) Enzalutamide-resistant VCaP cells were injected subcutaneously in male SCID mice, and once the tumors were palpable, the mice received oral gavage with either vehicle (6% Captisol; *n* = 10) or 24 (*n* = 10) or 48 mg/kg of (*R*)-**9b** (*n* = 10), five times a week. The tumor volumes were measured twice a week. Tumors were harvested once they reached ~1000 mm^3^ in volume. (**B** and **C**) The tumors were photographed (B), and their weights measured (C). (**D** to **F**) Tumor RNA was prepared, followed by qRT-PCR to determine expression of *AR* (**D**), *KLK3* (**E**), and *TMPRSS2* (**F**) mRNA (*n* = 3). Data are represented as means ± SEM. *P < 0.05, **P < 0.01, and ***P < 0.001, Student’s *t* test. (**G**) Tumor lysates (*n* = 3) were IP using either acK609-AR or pY267-AR antibodies, followed by immunoblotting with AR antibody (top two panels). Shown below each blot is the densitometric measurement of change in abundance relative to control.

To investigate whether (*R*)-**9b** can exert its tumor-suppressing effect when administered orally, enzalutamide-resistant VCaP cells were implanted subcutaneously in male SCID mice. When the tumors reached about 100 mm^3^ in size, the mice were randomized and received vehicle (6% Captisol) or (*R*)-**9b** (24 or 48 mg/kg of body weight) by oral gavage five times a week. (*R*)-**9b**– treated mice displayed a marked reduction in tumor volume and weight, as opposed to the robust tumor growth observed for vehicle-treated mice (Fig. 6, A to C). Of 10 mice that were gavaged with (*R*)-**9b** at 48 mg/kg, eight mice formed tumors, which were also markedly smaller (Fig. 6B). Similar to (*R*)-**9b** injection, oral gavage of (*R*)-**9b** also caused a marked reduction in *AR*, *KLK3*, and *TMPRSS2* mRNAs in comparison to the vehicle-treated group (Fig. 6, D to F). Furthermore, oral inoculation of (*R*)-**9b** also caused a reduction in pY284-ACK1, leading to the loss of pY267-AR and, hence, acK609-AR expression (Fig. 6G), providing insights regarding the probable mechanism of tumor growth inhibition upon (*R*)-**9b** treatment. These data provide evidence of feasibility of oral dosing of (*R*)-**9b**, emphasizing the importance of this route and formulation for future clinical trials.

### AR K609 acetylation is required for enzalutamide-resistant CRPC tumor growth

**Fig. 7.**
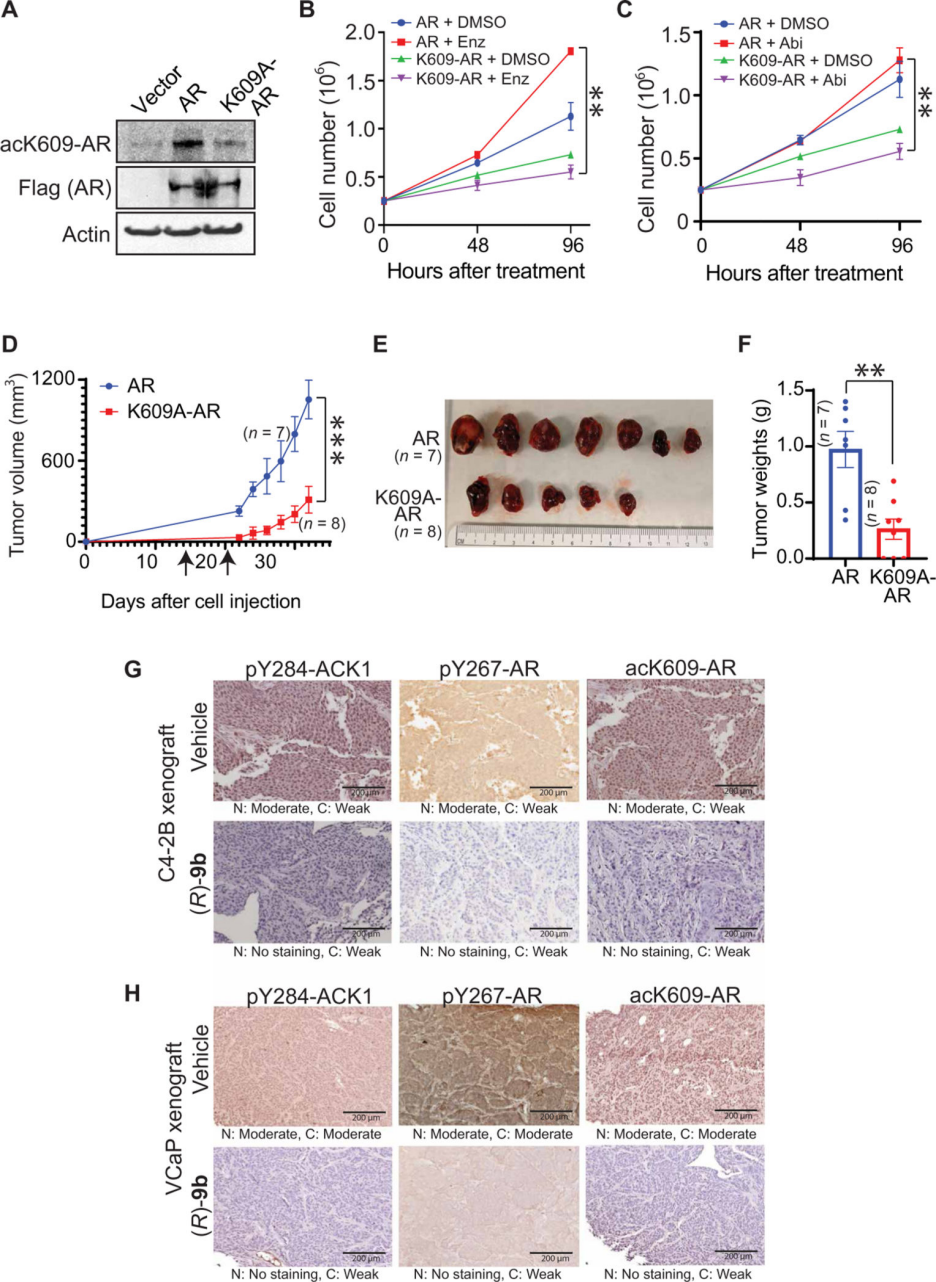
AR 609 acetylation is required for enzalutamide-resistant CRPC tumor growth. (**A**) C4–2B cells infected with a retroviral construct expressing FLAG-tagged AR or K609A-AR mutant were subjected to IP with acK609-AR antibodies, followed by immunoblotting with FLAG antibodies (top) or FLAG and actin antibodies (bottom). (**B** and **C**) Retrovirally infected C4–2B cells were grown in the presence of enzalutamide (Enz) (B) or abiraterone (Abi) (C), and cell proliferation was measured by trypan blue staining. (**D**) Enzalutamide-resistant C4–2B cells were infected with retrovirus-expressing AR or K609A mutant AR and injected subcutaneously in male SCID mice. Both sets of the mice were injected with enzalutamide (40 mg/kg) on days 17 and 23 after cell injection (shown by arrow). Tumor volumes were measured twice a week. (**E** and **F**) Once they reached ~1000 mm^3^ in volume, tumors were harvested, photographed, and weighed. Data are represented as means ± SEM. *P < 0.05, **P < 0.01, and ***P < 0.001, Student’s *t* test. (**G**) C4–2B and (**H**) VCaP xenograft tumors were treated with (*R*)-**9b** subcutaneously or orally, respectively, and were IHC-stained using pY284ACK1, pY267-AR, or acK609-AR antibodies (*n* = 3, per group; one representative image shown). Photographs were captured at ×20 magnification. N, nucleus; C, cytoplasm. Pathologist’s observation for intensity of the staining (range: no staining to moderate staining) is shown below the images.

To further pinpoint the role of AR K609 acetylation in promoting tumor growth, enzalutamide-resistant C4–2B cells were infected with a retroviral construct expressing FLAG-tagged K609A-AR mutant or AR (Fig. 7A). The K609A-AR mutant–expressing C4–2B cells exhibited decreased proliferation in enzalutamide- or abiraterone-rich media, whereas the acetylation-competent AR-expressing cells continued to grow (Fig. 7, B and C).

Moreover, when implanted subcutaneously in male SCID mice, K609A-AR mutant–expressing cells exhibited a marked decrease in tumor growth in the presence of enzalutamide (Fig. 7, D to F). Of eight mice that were injected with K609A-AR mutant–expressing cells, five mice formed tumors, which were also markedly smaller (Fig. 7E). In contrast, all the mice injected with AR-expressing cells formed robust tumors in the presence of enzalutamide (Fig. 7, D to F). Together, these data establish that AR K609 acetylation is a necessary event in CRPC for the acquisition of resistance to enzalutamide.

To verify the role of activated ACK1-mediated AR Y267 phosphorylation in K609 acetylation, (*R*)-**9b**–treated C4–2B and VCaP xenograft tumors (Figs. 5 and 6) were subjected to IHC staining. Decreased pY284-ACK1 staining was observed for (*R*)-**9b**–treated tumors, which was reflected in a suppression of pY267-AR and acK609-AR staining (Fig. 7, G and H).

### AR Tyr^267^ phosphorylation and K609 acetylation are up-regulated in human prostate cancer

**Fig. 8.**
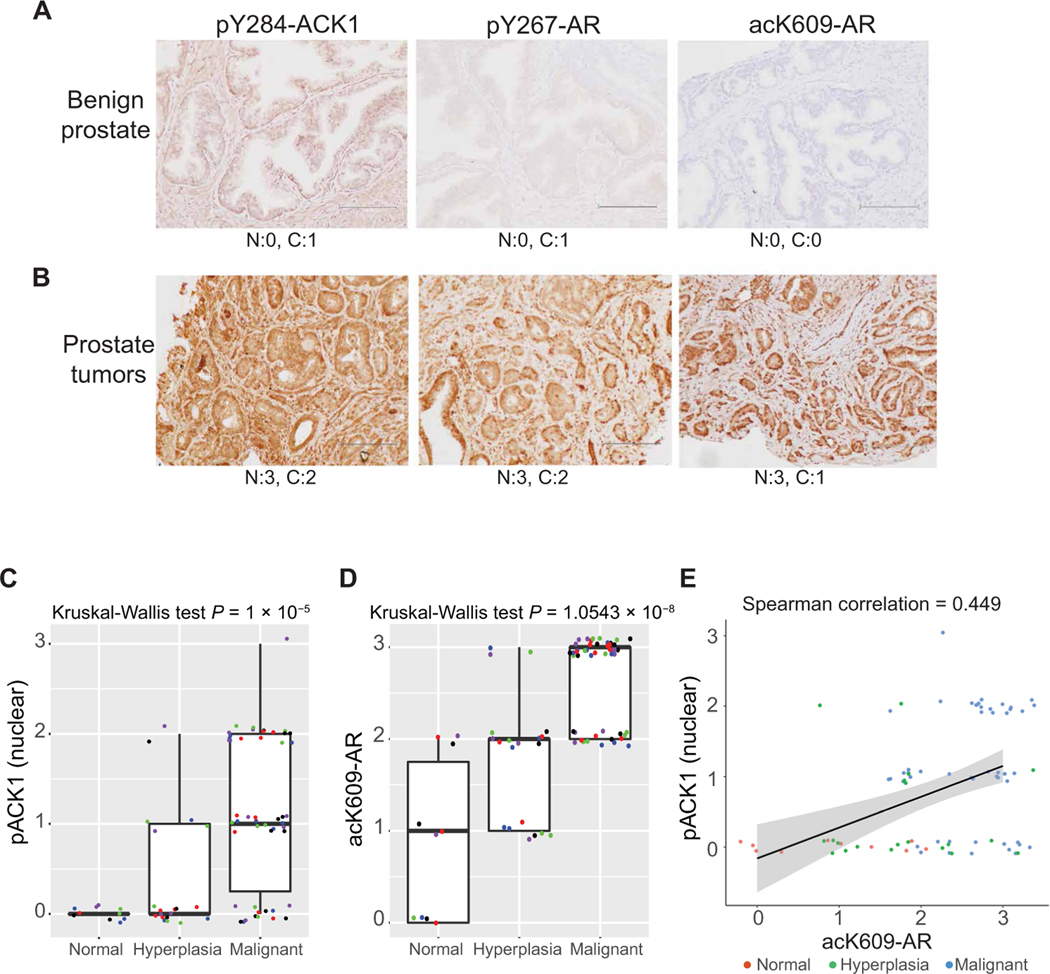
Enhanced AR 609 acetylation in enzalutamide-resistant CRPC tumors. (**A** and **B**) Human benign prostate tissues (A) and prostate tumors (B) were IHC-stained with pY284-ACK1, pY267-AR, or acK609-AR antibodies (*n* = 4 per group; one representative image shown). N, nucleus; C, cytoplasm. Pathologist’s scores for intensity of the staining (range: 0 to 4) are shown below the images. Bars represent 200 μm. (**C** and **D**) Box plots summarize distributions of staining intensities for pY267-AR and acK609-AR antibody in prostate tissue microarray sections. The box indicates 50% of the data from the 25% quartile to the 75% quartile with the bold black horizontal lines representing the median. Whiskers extend from each end of the box to the most extreme values within 1.5 times the interquartile range from the ends of the box. Individual data points were jittered and colored for better visualization. (**E**) Expression of pY267-AR and acK609-AR were significantly correlated in prostate tumors (ρ = 0.449, *P* = 2.9 × 10^−5^).

To examine the presence of acK609-AR in human prostate tumors, freshly harvested benign prostate tissues and a tumor from a patient with prostate cancer who was treated with enzalutamide were subjected to IHC staining. Increased pY284-ACK1, pY267-AR, and acK609-AR were observed in the tumors; in contrast, the benign prostate tissues exhibited markedly reduced staining for these three modifications (Fig. 8, A and B).

To examine whether ACK1 activation correlated with AR K609 acetylation in prostate cancer progression to metastatic stage, we performed tissue microarray analysis of clinically annotated prostate tumor samples. IHC analysis revealed increased expression of pY284-ACK1 and acK609-AR when progressive stages were examined (normal, hyperplasia, and malignant stages; Fig. 8, C and D). Spearman ranked correlation analysis revealed a significant correlation between pY284-ACK1 and acK609-AR (Spearman correlation = 0.449, *P* = 2.9 × 10^−5^) (Fig. 8E). Overall, dual-modified AR seems to be the crucial component in the initiation of a feed-forward loop that promotes CRPC self-preservation by constantly increasing AR/ACK1 expression, even in an antagonist- rich environment (fig. S14).

## DISCUSSION

In this report, we uncover a dual-modified AR engendered by cancer cells to maintain high AR and ACK1, overcoming the effect of AR antagonists. Because of successive deposition of these modifications and their interdependence, targeting the earliest event, AR Y267 phosphorylation, using an ACK1 kinase inhibitor not only suppressed AR K609 acetylation but also caused a global shutdown of many critical genes, including *AR* and *ACK1*.

The AR DBD (residues 556 to 623) is conserved in steroid hormone receptors. Lys^609^ acetylation occurs in the vicinity of the distal box region, which enables AR dimerization and DNA interaction (33), indicating that AR K609 acetylation strengthens binding to DNA. The fact that K609 is conserved in other steroid receptors signifies that K609 acetylation in cancer cells may play a broader role in exerting growth advantages. Recent findings have suggested that GR and PR are activated, up-regulating the transcription of androgen response genes while bypassing AR in the acquisition of resistance to second-generation antiandrogens (34–36). It would be interesting to investigate the status of K609 acetylation in the ER, GR, and PR hormone receptors and examine whether it provides any added benefits in prostate (and possibly breast) cancer progression to drug-resistant stages.

In the x-ray crystal structure of the AR DBD complexed with the ARE, the major driver of protein-DNA interaction is binding of the AR DNA recognition helix in the major groove of the ARE (33, 37). Located within the DNA binding/hinge region, acetylation of K609 would likely alter AR interaction with the phosphate backbone and the electrostatic surface of the AR DBD. K609 is implicated in the binding of the AR inhibitor pyrvinium to the protein-DNA complex (38, 39). K609, along N610 and P612, has been reported to mediate the interaction with pyrvinium, which appears to lock one conformation of the DNA binding/hinge region to prevent recruitment of RNA Pol II (40). We observed decreased RNA Pol II binding to the AR enhancer AREM1 in the K609A mutant AR. It is possible that K609 acetylation not only alters the AR interaction with the minor groove but also changes the conformational dynamics of the DNA binding/hinge region to facilitate interactions with RNA Pol.

The crystal structure of the N-terminal transactivation domain where Tyr^267^ is located is not available. CBP/P300- interacting transactivator with Glu/Asp- rich C-terminal domain 2 (CITED2) is a transcription cofactor that interacts with several other transcription factors and cofactors and is shown to have a role in multiple cellular processes, including proliferation, apoptosis, differentiation, migration, and autophagy (41). High-affinity binding of the transactivation domain of CITED2 with the cysteine-histidine–rich 1 (CH1) domain of p300/CBP is available (42). It suggests that the CH1 (also known as transcription adaptor putative zinc finger (TAZ)/ zinc-finger) domain of p300/CBP provides an adaptable protein-protein interaction scaffold for interaction with the transcription factors. These data suggests that the AR transactivation domain could potentially fold around CH1 domain of p300/CBP in a similar way. Together, these data indicate that Y267 phosphorylation of the AR within the N-terminal transactivation domain could promote its interaction with the CH1 domain of p300/CBP, then allowing the acetyltransferase activity of p300/CBP to modify K609 to alter DNA binding activity of the AR.

Other genes that are regulated by acK609-AR include single-minded homolog 2 (SIM2), which is known to be the highly up-regulated gene in prostate cancer (43), and its isoform SIM2-s, a marker for aggressive prostate cancer (44). The androgen-metabolizing enzyme HSD17B11 has been implicated in advanced prostate carcinoma (45). Zinc Finger RANBP2-type containing 3 (ZRANB3) is a translocase known to associate with polyubiquitinated proliferating cell nuclear antigen after replication stress, and its depletion renders cells sensitive to DNA-damaging agents (46). A disintegrin-like and metalloprotease domain with thrombospondin type I motifs-like 3 (ADAMTSL-3)/punctin-2 is a glycoprotein secreted in the extracellular matrix and found to be highly expressed in normal and malignant prostatic tissue (47). The regulation of these genes shed light onto the importance of dual-modified AR in prostate cancer and feasibility of (*R*)-**9b** as a desirable therapeutic modality.

It has been reported that AR is acetylated at K630/632/633 in the hinge region (between the DNA and ligand-binding domains) by p300 and P300/CBP-associated factor (PCAF), important for the ligand-dependent AR function (48, 49). However, in our search for AR modifications in enzalutamideand abiraterone-resistant CRPC, we identified an acetylated site, K609, located in the zinc finger. Unlike K630 and K632/633 acetylation, K609 acetylation imparted ligand-independent AR activation. p300 was identified as an acetyltransferase for K609 acetylation, indicating that the same enzyme activity is summoned in later stages of disease, yet directed at a different location to obtain a distinct physiological outcome. We observed that AR Y267 phosphoryl ation is needed for K609 acetylation, which suggests that pY267-AR recruits p300 to promote its autoacetylation in an androgen-deficient environment. However, as AR Tyr phosphorylation is low in androgen-r ich environments (7, 9, 27), it is likely that p300 recruitment and subsequent AR K609 acetylation are favored in CRPCs. Whether AR Y267 phosphorylation also has a role to play in AR K630/ 632/633 acetylation is not clear and remains to be determined.

The inability of the K609 acetylation–deficient mutant to optimally trigger AR transcription activity using rat probasin (PB) promoter in the presence of DHT suggests that ac-K609 also has a role in canonical AR activation. The androgen-regulated Probasin (PB) promoter region has transcription factor–binding sites for FoxA1, Oct-1, c-jun, and neurofibromatosis-related protein (NF1) (50). All these motifs were also present in acK609-AR ChIP-seq data. The ability of acK609-AR to recognize distinct sites and some canonical AR- binding sites does increase its repertoire of genes targeted for transcriptional activation; however, whether a specific epigenetic event or other transcriptional coactivator is involved in this process remains to be clarified.

Recently, mRNA sequencing of 550 primary and 120 mCRPC samples demonstrated that CBP and p300 mRNA expression is associated with the AR signature (51). Furthermore, CCS1477, an inhibitor of p300/CBP bromodomain, inhibits proliferation and decreases AR- and C-MYC–regulated gene expression, modulated KLK3/PSA concentration, and has shown promise for the treatment of patients with advanced prostate cancer. Unlike HATs, there is no clear consensus in HDACs expression and prostate cancer; as a single agent, HDAC inhibitors have shown poor activity in CRPC and other solid tumors. Suberoylanilide hydroxamic acid (SAHA), depsipeptide (romidepsin), pracinostat (SB939), and panobinostat all failed in phase 2 clinical trials for prostate cancer (52). Together, these results support our data that the targeting of p300 and CBP as a means of blocking ac-AR signaling is beneficial in prostate cancer; however, inhibition of HDACs is likely to promote AR acetylation, proving to be counter-effective for patients.

Coordinated phosphorylation/acetylation modifications are also reported for the regulation of DNA binding by transcription factors, such as STAT proteins, however, with different consequences (53). Acetylation of STAT1 at K410 detaches STAT1 from chromatin, and STAT1 with a K410R mutation is more active than wild-type STAT1. Furthermore, acetylation of STAT1 inhibited interferon-dependent STAT1 phosphorylation and nuclear translocation. Moreover, ubiquitin specific peptidase 12 (USP12) inhibited CBP- mediated acetylation and maintained nuclear p-STAT1 and subsequent dephosphorylation of p-STAT1 in the nucleus (54). Thus, in contrast to phospho-acetyl switch that inhibits STAT1 transcription activity upon its acetylation, AR seems to use both the modifications to allow prostate cancer to evolve through progressive stages to become CRPC.

Our study has some limitations. Compared to AR, whether acK609-AR forms a distinct transcription complex is not clear. A common set of motifs recognized by acK609-AR in both enzalutamide-resistant VCaP and LNCaP cells indicates that CRPCs sense loss of AR activity, promoting a distinct transcription program using acK609-AR. How prostate cancer cells summon HATs to acetylate AR in response to AR antagonists is not clear and will be the topic of future investigation. In addition, we have not compared direct AR and acK609-AR DNA binding to chromatin in the absence of its ligand, DHT, which would be indicative of relative binding efficiency in AR antagonist–rich environment. Moreover, we have not yet tested the effect of (*R*)-**9b** in organoids derived from CRPCs and enzalutamide-resistant PDX tumors, which is a prerequisite for a prospective clinical trial of ACK1 inhibitors.

Overall, the “dual-modified” AR allows prostate cancer to evolve through two distinct therapy resistances, androgen deprivation, and AR antagonism. Identification of an ACK1 kinase inhibitor that has the ability to thwart both the modifications thus opens a therapeutic modality for patients with recurrent CRPC, a currently unfulfilled need.

## MATERIALS AND METHODS

### Study design

The objective of this study was to investigate how CRPCs develop resistance to the currently used therapy enzalutamide. Mass spectrometry, drug affinity chromatography, IP, immunoblotting, ChIP-seq, qRT-PCR, and other assays were used to explore how modified AR plays a role in imparting resistance. We obtained tumor samples from the Siteman Cancer Center and Barnes Jewish Hospital, Washington University in St Louis. Informed consent was obtained from all the participants, and the procedure was approved by the institutional review board (IRB). For xenograft studies, mice were randomly assigned to groups before initiating the experiment, examined daily, and humanely euthanized at defined study end points. Experimenters were not blinded to group allocation. All animal procedures were conducted in accordance with the guidelines of the Institutional Animal Care and Use Committee (IACUC) of Washington University in St Louis. For other assays, at least two or three independent experiments were performed, each with triplicates, with a representative study shown.

### Generation of acK609-AR monoclonal antibody

The antibody expressing clones were custom-generated by ProMab. Briefly, two AR peptides coupled to immunogenic carrier proteins were synthesized, an acetyl peptide: TIDKFRR[**K-ac**]NCPSCRL and a non-acetyl peptide: TIDKFRRKNCPSCRL. Five mice were immunized four times with acetyl peptides, several weeks apart, and an enzyme-linked immunosorbent assay was performed to determine the relative titer of sera against acetylated and non-acetylated peptides. The mice with the best titer (and least reactivity with non-acetylated peptide) were chosen for fusions. The supernatants of the clones were tested against peptides and immunoblotting of proteins. Two clones were selected, 5B9E4 (#7) and 5B9E5 (#8). The 5B9E4 (#7) clone supernatant was primarily used in this study.

### Human subjects

Patients with localized prostate cancer, metastatic hormone-sensitive prostate cancer, or mCRPC were recruited for the study. Prostate tumor and adjacent benign tissues from an enzalutamide-treated patient (*n* = 1) were assessed for AR acetylation/phosphorylation and ACK1 phosphorylation. Patients were consented to our IRB-approved genitourinary banking protocol [Human Research Protection Office (HRPO) no.: 201411135] to allow tissue collection. Tissues were fixed and sectioned, and hematoxylin and eosin staining was performed followed by pathologist review to determine the status of samples. IHC staining was performed for the tissue sections at the following concentration: acK609-AR (1:1), pY267-AR (1:50), and pY284-ACK1 (1:300). Pathologist’s (C.W.) scores for intensity of the staining (range: 0 to 4) are shown below the images.

### Resources

Sequences for the primers are in table S2. Sources of biochemicals, antibodies, cell lines, and software are in table S3.

### Mouse xenograft studies

All animal experiments were performed using the standards for humane care in accordance with the National Institutes of Health (NIH) *Guide for the Care and Use of Laboratory Animals*. Mice studies were performed according to IACUC protocols approved in writing by Washington University in St. Louis Department of Comparative Medicine (IACUC protocol nos. 20180247 and 20180259). C4–2B cells (2 × 10^6^) were suspended in 200 μl of PBS with 50% Matrigel (BD Biosciences) and were implanted subcutaneously into the dorsal flank of castrated 6-week-old male SCID C.B17 mice (*n* = 7 per group). Once the tumors become palpable (about 100 mm^3^ in size; about 4 to 5 weeks), mice were injected subcutaneously with (*R*)-**9b** resuspended in 6% Captisol (or 6% Captisol in PBS as vehicle) at the concentrations 12 and 20 mg/kg of body weight, five times a week, for 4 to 5 weeks. Tumor volumes were measured twice a week using calipers. Formation of tumors was monitored over a 10-week period. At the end of the study, all mice were humanely euthanized and tumors were extracted and weighed.

For the oral efficacy study, a similar strategy was used except that the VCaP cells were injected in male SCID C.B17 mice (*n* = 10 per group) and treated orally with 24 and 48 mg/kg of body weight with (*R*)-**9b** in 6% Captisol (or 6% Captisol in PBS as vehicle) five times a week. IHC staining was performed for the tumor sections as described above.

### Statistical analysis

Multiple statistical methods were used in this investigation depending on the data type. Data were expressed as means ± SEM. Data between two groups were analyzed with unpaired Student’s *t* tests using GraphPad Prism. A *P value of ≤0.05 was considered statistically significant. Before applying parametric tests such as Student’s *t* test, we visualized the distribution of data by plotting histograms, density, and quantile-quantile plots.

For the tissue microarray analysis, Kruskal-Wallis tests were performed to examine differences in nuclear pY284-ACK1 and acK609-AR with respect to different progression stages of prostate cancer. Boxplots were used to summarize the intensity distribution at each progression stage. Analysis of variance (ANOVA) was done to examine differences in nuclear expression ofpTyr284-ACK1 and acK609-AR at different tumor stages, and Tukey’s method was performed to adjust for all pairwise comparisons. Correlations were done using Spearman rank-based correlation. All analyses were conducted using GraphPad Prism (GraphPad Software Inc.) and R (version 4.1.1; http://cran.r-project.org). All tests were two sided, and statistical significance was defined at 5% alpha.

## Data and materials availability:

All data associated with this study are present in the paper or the Supplementary Materials. Raw data can be found in data files S1 to S3. ChIP-seq data are available at the National Center for Biotechnology Information Gene Expression Omnibus (NCBI GEO) accession number GSE162761. All data accessed from external sources and prior publications have been referenced in the text and corresponding figure legends. A small-molecule inhibitor of ACK1, (*R*)-**9b**, can be made available through a material transfer agreement with Moffitt Cancer Center, Tampa, FL.

## Supplementary Material

SI information Sci Tranl Med
