# Supplementary material for: Chronologically modified androgen receptor in recurrent castration-resistant prostate cancer and its therapeutic targeting
Source: Sci Transl Med. Author manuscript; Available in PMC 2023 Jun 12. (PMC10259236; doi:10.1126/scitranslmed.abg4132)
Supplement: SI information Sci Tranl Med [file NIHMS1861517-supplement-SI_information_Sci_Tranl_Med.pdf]

Science Translational Medicine
AAAS

Supplementary Materials for

Chronologically modified androgen receptor in recurrent castration-resistant prostate cancer and its therapeutic targeting

Mithila Sawant *et al.*

Corresponding author: Nupam P. Mahajan, nupam@wustl.edu

Sci. Transl. Med. **14**, eabg4132 (2022)
DOI: 10.1126/scitranslmed.abg4132

The PDF file includes:

Materials and Methods
Figs. S1 to S14
Tables S1 to S3
References (*55–64*)

Other Supplementary Material for this manuscript includes the following:

Data files S1 to S3
MDAR Reproducibility Checklist

Materials and Methods

Cell lines, antibiotics, inhibitors, and media

LNCaP, VCaP, PC3, 22Rv1, DU145 and MCF7 cells were obtained from ATCC and have grown as per ATCC instructions. All cultures were maintained with 50 units/ml of penicillin/streptomycin (Invitrogen) and cultured in 5% CO2 incubator. Although cells were grown in FBS containing media, for most experiments, cells were grown in media with charcoal stripped fetal bovine serum (CS-FBS) to deplete androgen and then treatment were performed. RGFP966, enzalutamide and abiraterone were obtained from Targetmol, puromycin from Sigma, and polybrene from Gibco. Inhibitors C646 and SBHA were obtained from Sigma. Captisol was obtained from CyDex Pharmaceuticals, Inc.

Drug affinity chromatography

The enzalutamide-biotin conjugate bound streptavidin sepharose beads (at the final drug concentration of 25 nmol/50 µl beads) were incubated with the nuclear lysates prepared from enzalutamide-resistant prostate cancer cells, C4-2B. The beads linked to (*R*)-**9b** were used as negative control. Beads were washed and bound proteins were eluted, electrophoresed, followed by mass spectrometry.

Linking enzalutamide and (*R*)-9b to biotin

Biotinylated (*R*)-**9b** was prepared by replacing its *N*-methyl group with the polyethylene-based linker conjugate *N*-(13-*N*-succinimidyl-4,7,10-trioxatridecanyl)biotinamide (*56*) (biotin-X-CO_2H) (**fig. S1 and S2**). The *N*-methyl group of (*R*)-**9b** is oriented towards solvent when bound to ACK1 and is therefore a suitable group for substitution with the biotin-linker without affecting affinity for ACK1 (*57*). Similarly, biotinylated enzalutamide was prepared by attaching the same biotin reagent biotin-X-CO_2H to an aminoethylene amide derivative of its *N*-methyl amide. Structure activity studies have revealed that groups larger than the methyl amide at that position, are well tolerated (*58*). Recently, PROTAC reagents linked to an E3-ligase binder through this position effectively degrade AR (*59*), providing further support that AR binding affinity is retained. Additionally models proposed for enzalutamide-AR binding, suggest that the methyl amide group

is positioned towards solvent, and therefore biotin-enzalutamide should have similar affinity for AR as enzalutamide itself (*60*).

General Chemistry Experimental Information. The description of the origin, use and handling of chemical reagents is the same as we have reported previously (*57*). The preparative HPLC used to purify the biotin-linked probes was carried out using ZORBAX Eclipse XDB 80 Å C18, 21.2 × 250 mm cartridge, 7 µm and JASCO HPLC system equipped with a PU-2089 Plus quaternary gradient pump and a UV-2075 Plus UV-VIS detector.

Synthesis of Biotin-(*R*)-9b (fig. S2): Intermediate **3** was prepared by the synthetic route shown in fig. S2 which we have reported previously (*57*). The Biotin-X-CO_2H was synthesized using a literature reported protocol (*56*). A mixture of Biotin-X-CO_2H (*56*) (55.0 mg, 0.1 mmol), 1*H*-1,2,3-benzotriazol-ol (HOBt) (20.3 mg, 0.15 mmol), 1-ethyl-3-(3-dimethylamino-propyl)carbodiimide (EDC) (29.0 mg, 0.15 mmol) and diisopropylethylamine (104.5 µL, 0.6 mmol) in dichloromethane (1.0 mL) and *N*,*N*-dimethylformamide (1.0 mL) was stirred 1 h at room temperature, and added intermediate **3** (65.0 mg, 0.105 mmol). The reaction was stirred at room temperature for 18 h and monitored using HPLC-MS. The HPLC-MS showed the formation of the desired product. The reaction mixture was diluted with ethyl acetate (15 mL), cloudy mixture obtained, added 3 drops of methanol to obtain a clear mixture. This mixture was washed with water (5 mL), 1M hydrochloric acid (3 mL), and brine (3 mL). The organic phase showed no product. The aqueous phase was evaporated to dryness, and the solid obtained was filtered and washed with methanol. The filtrate was dried, added methanol (2 mL) to obtain crude product as white solid. The crude product was triturated with ethyl acetate (1-2 mL) to remove impurity, and the oil obtained was purified using reverse phase preparative HPLC using methanol:water with 0.1% TFA, 35%- 65% gradient elution, 20 ml/min, 60 min. The desired product was eluted around 27-30 min, and the fractions containing the product were concentrated and dried in a lyophilizer to obtain **Biotin (*R*)-9b** as a white solid (38 mg, 30%). HPLC; 96.7% [t_R = 5.14 min, Grad. MeOH/water (50:50, with 0.1% formic acid) 20 min]; ^{1}H NMR (400 MHz, CD_3OD) δ 9.75 (brs, 1H), 8.19 (brs, 1H), 8.0 (s, 1H), 7.81 (t, *J* = 5.2 Hz, 1H), 7.75 (t, *J* = 5.6 Hz, 1H), 7.41 (d, *J* = 8.0 Hz, 2H), 6.96 (d, *J* = 8.8 Hz, 2H), 6.42 (s, 1H), 6.35 (brs, 1H), 4.27 (dd, *J* = 7.6, 4.8 Hz, 1H), 4.11-

4.01 (m, 1H), 3.70 (q, J = 6.8 Hz, 1H), 3.62-3.58 (m, 5H), 3.50-3.34 (m, 10H), 3.38-3.34 (m, 5H), 3.12-3.09 (m, 8H), 2.78 (dd, J = 12.8, 5.2 Hz, 1H), 2.57-2.53 (m, 3H), 2.31-2.28 (m, 3H), 2.03-1.99 (m, 2H), 1.86-1.76 (m, 4H), 1.61-1.53 (m, 5H), 1.46-1.41 (m, 3H), 1.29-1.25 (m, 2H); HPLC-MS (ESI) *m/z* 917.4 (M+H, 20%)$^+$, 459.4 [(M+2H), 100%]$^{2+}$; HRMS (ESI+) *m/z* calculated for $C_{43}H_{65}ClN_{10}O_8S$ (M+H)$^+$ 917.4487, found, 917.4469.

Synthesis of Biotin-enzalutamide (fig. S2).

tert-Butyl (2-(4-(3-(4-cyano-3-(trifluoromethyl)phenyl)-5,5-dimethyl-4-oxo-2-thioxoimidazolidin-1-yl)-2-fluorobenzamido)ethyl)carbamate (**compound 10**): A mixture of *tert*-butyl (2-aminoethyl)carbamate (84.0 mg, 0.52 mmol), 1*H*-1,2,3-benzotriazol-ol (HOBt) (101.0 mg, 0.75 mmol), 1-ethyl-3-(3-dimethylamino-propyl)carbodiimide (EDC) (144.0 mg, 0.75 mmol) and diisopropylethylamine (0.523 µL, 3.0 mmol) in dichloromethane (2.0 mL) and *N,N*-dimethylformamide (1.0 mL) was stirred 1 h at room temperature. To this mixture was added enzalutamide acid **9** (*61*)(226.0 mg, 0.5 mmol), prepared according to the synthetic route shown in fig. S2, by similar methods to those reported (*62*). The reaction was stirred at room temperature for 42 h and monitored using HPLC-MS, and HPLC-MS showed a mixture of the required product and SM. The reaction was stopped, and the *N,N*-dimethylformamide was removed using a rotary evaporator. The residue was purified using SiO_2 chromatography using EtOAc/hexane gradient elution, and the product was eluted with 40% EtOAc in hexane. The NMR and HPLC-MS analysis indicated the presence of approximately 10% HOBt, and this mixture (130 mg, 44%) was used in the next step without further purification. ^{1}H NMR (400 MHz, DMSO-d_6) δ 8.47 (broad t, J = 5.6 Hz, 1H), 8.39 (d, J = 8.4 Hz, 1H), 8.27 (d, J = 1.6 Hz, 1H), 8.06 (dd, J = 8.4, 1.6 Hz, 1H), 7.78 (t, J = 8.4 Hz, 1H), 7.41 (dd, J = 10.8, 2.0 Hz, 1H), 7.31 (dd, J = 8.4, 2.0 Hz, 1H), 6.90 (t, J = 5.6 Hz, 1H), 3.26 (q, J = 6.0 Hz, 2H), 3.09 (q, J = 6.0 Hz, 2H), overlapping the HOD signal, 2H), 2.69 (t, J = 6.8 Hz, 2H), 1.52 (s, 6H), 1.36 (s, 9H); ^{19}F NMR (376 MHz, DMSO-d_6) δ -60.79 (s, 3F), -112.40 (t, $^3J_{HF}$ = 9.4 Hz, 1F); HPLC-MS (ESI+) *m/z* 538.1 (M-^{t}Bu+2H, 100%)$^+$, 494.2 (M-Boc+2H, 60%)$^+$.

N-(2-aminoethyl)-4-(3-(4-cyano-3-(trifluoromethyl)phenyl)-5,5-dimethyl-4-oxo-2-thioxoimidazolidin-1-yl)-2-fluorobenzamide (**compound 11**): To compound **10** (130 mg, 0.219 mmol) in dichloromethane (2.0 mL) at 0 ºC, was added trifluoroacetic acid (TFA) (2.0 mL). The reaction was warmed to room temperature and stirred for 2 h. The solvent was removed, and the residue obtained was redissolved in dichloromethane (20.0 mL), washed with sat. sodium bicarbonate (20.0 mL), and brine (10.0 mL). The organic phase was separated, dried (Na_2SO_4) and concentrated to dryness to afford compound **11** (86 mg, 80%) as a yellow oil. This product was carried to the next stage without further purification. ^{1}H NMR (400 MHz, DMSO-d_6) δ 8.47 (broad t, *J* = 5.2 Hz, 1H), 8.39 (d, *J* = 8.4 Hz, 1H), 8.27 (d, *J* = 1.6 Hz, 1H), 8.06 (dd, *J* = 8.4, 1.6 Hz, 1H), 7.81 (t, *J* = 8.4 Hz, 1H), 7.41 (dd, *J* = 10.8, 2.0 Hz, 1H), 7.31 (dd, *J* = 8.4, 2.0 Hz, 1H), 3.28-3.25 (m overlapping the HOD signal, 2H), 2.69 (t, *J* = 6.8 Hz, 2H), 1.52 (s, 6H); ^{19}F NMR (376 MHz, DMSO-d_6) δ -60.79 (s, 3F), -112.57 (t, $^3J_{HF}$ = 9.0 Hz, 1F); HPLC-MS (ESI+) *m/z* 494.2 $(M+H, 100\%)^+$.

Biotin-enzalutamide (fig. S2): A mixture of Biotin-X-CO_2H (*56*) (68.0 mg, 0.125 mmol), 1*H*-1,2,3-benzotriazol-ol (HOBt) (25.4 mg, 0.188 mmol), 1-ethyl-3-(3-dimethylamino-propyl)carbodiimide (EDC) (36.0 mg, 0.188 mmol) and diisopropylethylamine (131.0 µL, 0.75 mmol) in dichloromethane (1.0 mL) and *N,N*-dimethylformamide (1.0 mL) was stirred 1 h at room temperature. To this mixture was added intermediate amine **11** (65.0 mg, 0.105 mmol). The reaction was stirred at room temperature for 48-50 h when HPLC-MS confirmed conversion to the desired product. The solvent was removed using an Agilent V-10 evaporator, and the crude material was purified using preparative HPLC using methanol:water with 0.1% TFA, 55-80% gradient elution, 20 ml/min, 60 min. The desired product eluted around 14.5-15.5 min. The fractions containing the product were concentrated and dried in a lyophilizer to obtain **Biotin-enzalutamide** as a white solid (57 mg, 45%). HPLC 99.5% [t_R = 7.97 min, 60% CH_3OH in 0.1% TFA water 20 min.]; ^{1}H NMR (400 MHz, DMSO-d_6) δ 8.50 (broad t, *J* = 5.2 Hz, 1H), 8.39 (d, *J* = 8.4 Hz, 1H), 8.27 (d, *J* = 1.6 Hz, 1H), 8.06 (dd, *J* = 8.4, 1.6 Hz, 1H), 7.95 (broad t, *J* = 5.6 Hz, 1H, disappeared upon D_2O shake), 7.83-7.73 (m, 3H which upon D_2O shake became t, *J* = 8.4 Hz, 1H), 7.41 (dd, *J* = 10.8, 2.0 Hz, 1H), 7.31 (dd, *J* = 8.4, 2.0 Hz, 1H), 4.29-4.26 (m, 1H), 4.11-4.08 (m, 1H), 3.50-3.42 (m, 12H), 3.20-3.18 (m, 2H), 3.09-3.01 (m, 6H), 2.79 (d, *J* = 12.4, 5.2 Hz, 1H),

2.28 (s, 4H), 2.02 (t, J = 6.8 Hz, 2H), 1.59-1.40 (m, 10H overlapping a sharp s, 6H), 1.30-1.25 (m, 2H); ^{19}F NMR (376 MHz, DMSO-d_6) δ -60.79 (s, 3F), -112.35 (t, $^3J_{HF}$ = 9.0 Hz, 1F); HPLC-MS (ESI+) *m/z* 511.6 (M+H, 100%)$^{2+}$; HRMS (ESI+) *m/z* calculated for $C_{46}H_{60}F_4N_9O_9S_2^+$ (M+H)$^+$ 1022.3886, found, 1022.3856.

AR acetyl-Site Determination Using Mass Spectrometry

Following affinity purification with streptavidin-beads, purified AR was subjected to SDS PAGE electrophoresis and the gel was stained Coomassie Brilliant Blue R250 (BioRad). A prominent band of 101 kDa was excised, washed once with water and twice with 50 mM ammonium bicarbonate in 50% aqueous methanol. Proteins were reduced and alkylated with 2 mM Tris(2-carboxyethyl)phosphine hydrochloride (TCEP) (Sigma) and 20 mM iodoacetamide (GE Healthcare), respectively. Samples were digested overnight with modified sequencing grade trypsin (Promega), Glu-C (Worthington), or chymotrypsin (Roche). Peptides were extracted from the gel slices. A nanoflow liquid chromatograph (Ultimate3000) coupled to an electrospray hybrid ion trap mass spectrometer (LTQ Orbitrap) was used for tandem mass spectrometry peptide sequencing experiments. Peptides were separated with a C18 reverse phase column (LC Packings C18 Pepmap) using a 40 min gradient from 5%B to 50%B (B: 90% acetonitrile/0.1% formic acid). The flow rate on the analytical column was 300 nl/min. Five tandem mass spectra were acquired for each MS scan using 60 sec exclusion for previously sampled peptide peaks (Spray voltage 2.3 kV, 30% normalized collision energy, scanning m/z 450–1,600). Sequences were assigned using Sequest (Thermo) and Mascot (www.matrixscience.com) database searches against SwissProt protein entries of the appropriate species. Acetylated lysines were selected as variable modifications, and as many as 3 missed cleavages were allowed. The precursor mass tolerance was 1.08 Da and MS/MS mass tolerance was 0.8Da. Assignments were manually verified by inspection of the tandem mass spectra and coalesced into Scaffold reports (www.proteomesoftware.com).

Proliferation Assay

To assess the effect of various treatments on cell proliferation, the cells were treated with Vehicle (DMSO), Enzalutamide and Abiraterone in complete media for 7 days and number of viable cells counted by trypan blue exclusion assay. For examining proliferation of retro-viral infected cells,

the cells were plated after puromycin selection (0.5ug/10ml) ($2.5x10^5$ cells per well of 6 well plate), treated with DMSO or Enzalutamide for 24h, 48h and 96h and the cell viability was estimated using Trypan blue exclusion assay.

Transfections and Retro-viral Infections

HEK293, DU145, MCF7, PC3 cells were transfected with either of the plasmids cDNA3.1, WT-AR, mutants AR (K609A), AR (K609Q) or ACK1 according to the experiments performed using X-tremeGENE-HP Transfection (Roche) reagent according to manufacturer's protocol. The cells were harvested 48 hours post-transfection and processed. For retro-viral infection, HEK293T cells were used and transfected along with gag, pol and env expressing plasmids as mentioned above. The supernatant was collected after 48h, 72h and 96h post-transfection and incubated with either VCaP or C42B cells with 8ug/ml polybrene (Sigma). The cells were selected using 1.5ug puromycin for VCaP and 0.5ug puromycin for C4-2B cells overnight.

Western Blot Analysis

LNCaP, VCaP, LNCaP-C4-2B, HEK-293 or PC3 cells were grown in at $5x10^6$ cells/ml. For AR, ACK1, Actin, Histone H4, HA and FLAG detection, treated cells were harvested and lysed by sonication in receptor lysis buffer (RLB) (*55*). After blocking in 5% nonfat dry milk (or 3% BSA), membranes were incubated with the following primary antibodies: AR mouse monoclonal antibody (1:1000; Santacruz), ACK1 mouse monoclonal antibody (1:1000; Santacruz), pACK1 mouse monoclonal antibody (1:1000; Upstate), Actin mouse monoclonal antibody (1:10,000; Sigma), Histone H4 mouse monoclonal antibody (1:3000; Cell Signaling) or HA mouse monoclonal antibody (1:2000; Santacruz) and FLAG mouse monoclonal antibody (1:4000; Sigma). The blots were washed and the signals visualized by enhanced chemiluminescence (ECL) system according to manufacturer's instructions (GE Healthcare).

For detection of acK609-AR, cell lysates were quantitated and 1.5-2 mg of protein lysate was immunoprecipitated using 3-4 µl of acK609-AR antibody coupled with protein A/G-sepharose (Santacruz) overnight, followed by immunoblotting was performed using anti-AR antibody as described above.

For Dot Blot analysis, 20ng of acK609-AR peptide and 20ng of corresponding non-acetylated peptide were blotted onto nitrocellulose membrane, followed by blocking in 3% BSA, membranes were incubated with individual primary acK609-AR monoclonal antibodies overnight. After washes, the blots were incubated with HRP-conjugated secondary antibody. In order to visualize the total protein, control blots were stained using Ponceau S staining solution [0.1% Ponceau S (w/v) and 5.0% acetic Acid (w/v)].

Chromatin Immunoprecipitation (ChIP) and ChIP-Sequencing

For ChIP-Seq, VCaP cells ($5x10^7$ cells) were treated with vehicle or (*R*)**-9b,** and LNCaP cells were treated with Vehicle or Enzalutamide. Cell pellets were resuspended in RLB buffer and sonicated for 25 seconds. The soluble chromatin was incubated overnight at 4°C with antibodies and protein-G/A magnetic beads. Ten nanograms of immunoprecipitated DNA was used to generate sequencing libraries using the Kapa Hyper Prep Kit (Roche Sequencing Solutions Inc.). The size and quality of the library was evaluated using the Agilent BioAnalyzer (Agilent Technologies, Inc.), and the library was quantitated with the Kapa Library Quantification Kit. Each enriched DNA library was then sequenced on an Illumina NextSeq 500 sequencer to generate 40-50 million 75-base paired-end reads (Illumina, Inc.). The raw sequence data were aligned using BowTie 2 (*63*), and the binding sites were identified using the MACS peak-finding software (*64*).

For validation, ChIP was performed using either acK609-AR, p300 (Santacruz), FLAG, ACK1 or RNA Pol II (Active Motif) antibody or IgG (Sigma). The amount of immunoprecipitated DNA was determined by real-time PCR, as described below.

Bioinformatic analysis

Raw fastq files were aligned against human genome hg38 assembly using Burrows-Wheeler Alignment tool and then processed using methylQA to generate bed and bigwig files. The MACS2 peak caller was used to compare the ChIP-Seq signal to a corresponding input control to identify narrow regions of enrichment (peaks) using default parameters. ChIP-Seq signals and peak locations were further visualized using UCSC Genome Browser and IGV software. The top 500-1000 ChIP-Seq peaks were assigned to the nearest genes using the annotatePeaks function from HOMER2, motif binding sites were determined using HOMER2, and GO and pathway enrichment

analyses were performed by EnrichR. The ChIP-seq data was deposited; the GEO accession is GSE162761. For comparison, AR ChIP-Seq data from VCaP cells were downloaded from GEO datasets (GSE28950; GSM717391 for Input, and GSM717392 for AR ChIP-Seq).

Quantitative RT-PCR and ChIP-qPCR

For the construction of standard curves, serial dilutions of pooled sample RNA were used (50, 10, 2, 0.4, 0.08, and 0.016 ng) per reverse transcriptase reaction. One ‘‘no RNA’’ control and one ‘‘no Reverse Transcriptase’’ control was included for the standard curve. Three reactions were performed for each sample: 10 ng and a NoRT (10 ng) control. Real-time quantitative PCR analyses were performed using the ABI PRISM 7900HT Sequence Detection System (Applied Biosystems). Dissociation curves were generated for each plate to verify the integrity of the primers. Data were analyzed using SDS software version 2.2.2 and exported into an Excel spreadsheet. The 18S data were used for normalizing the gene values. Sequences for the primers are shown in **table S2**.

For ChIP-QPCR, Serial dilution of 2µl pooled input DNA from all the samples was used to construct the standard curve (1:1, 1:5, 1:25, 1:125 and 1:625). The input DNA was diluted 1:5 for all the samples. 2µl of undiluted immunoprecipitated DNA and diluted Input DNA was used for each sample.

Tissue Microarray Analysis

We extensively validated acK609-AR antibodies for the IHC staining in prostate tissues using various samples (positive and negative controls) at different antibody concentrations. Negative controls were included by omitting acK609-AR antibody during primary antibody incubation or HRP labelled secondary antibody to negate background. VCaP, LNCaP, LaPC4, and C4-2B cells treated with either vehicle or (*R*)-**9b** were fixed, encapsulated in HistoGel (Fisher Scientific) paraffin-embedded, sectioned, and used for antibody validation. The IHC staining conditions for pY284-ACK1 were published previously (*55*).

For assessment of pY284-ACK1 and acK609-AR expression, immunohistochemistry was carried out on TMA (n = 80 cores). The tissue array slides (including positive and negative controls)

were stained for the antibodies. The slides were dewaxed by heating at 65° Celsius for 60 min, washed two times, 15 min each, with xylene. Tissues were rehydrated by two series of 10 min washes in 100%, 95%, and 70% ethanol and distilled water. Antigen retrieval was performed by heating the samples at 95^0C for 25 min in 10 mmol/L sodium citrate (pH 6.0). The slides were cooled in PBS for 30 min, with 10 min changes of PBS and permeabilized using 0.2% Triton-X100 in PBS for 10 min. Slides were washed with PBS for 10 min. After blocking with universal blocking serum (DAKO Diagnostic) for 30 min, the samples were incubated with rabbit polyclonal pY284-ACK1 antibody (1:300 dilution; Millipore) and mouse monoclonal acK609-AR antibody (1:1 dilution) at 4^0C overnight. The sections were incubated with biotin-labeled secondary and streptavidin-peroxidase for 30 min each (DAKO Diagnostic). The samples were developed with 3,39-diaminobenzidine substrate (Vector Laboratories) and counterstained with hematoxylin. Following standard procedures, the slides were dehydrated and sealed with cover slips. The pY284-ACK1 and acK609-AR staining in paraffin embedded tissues were examined in a blinded fashion by pathologist (C.W.). Positive reactions were scored into four grades according to the intensity of staining: 0, 1+, 2+ and 3+.

Figure S1

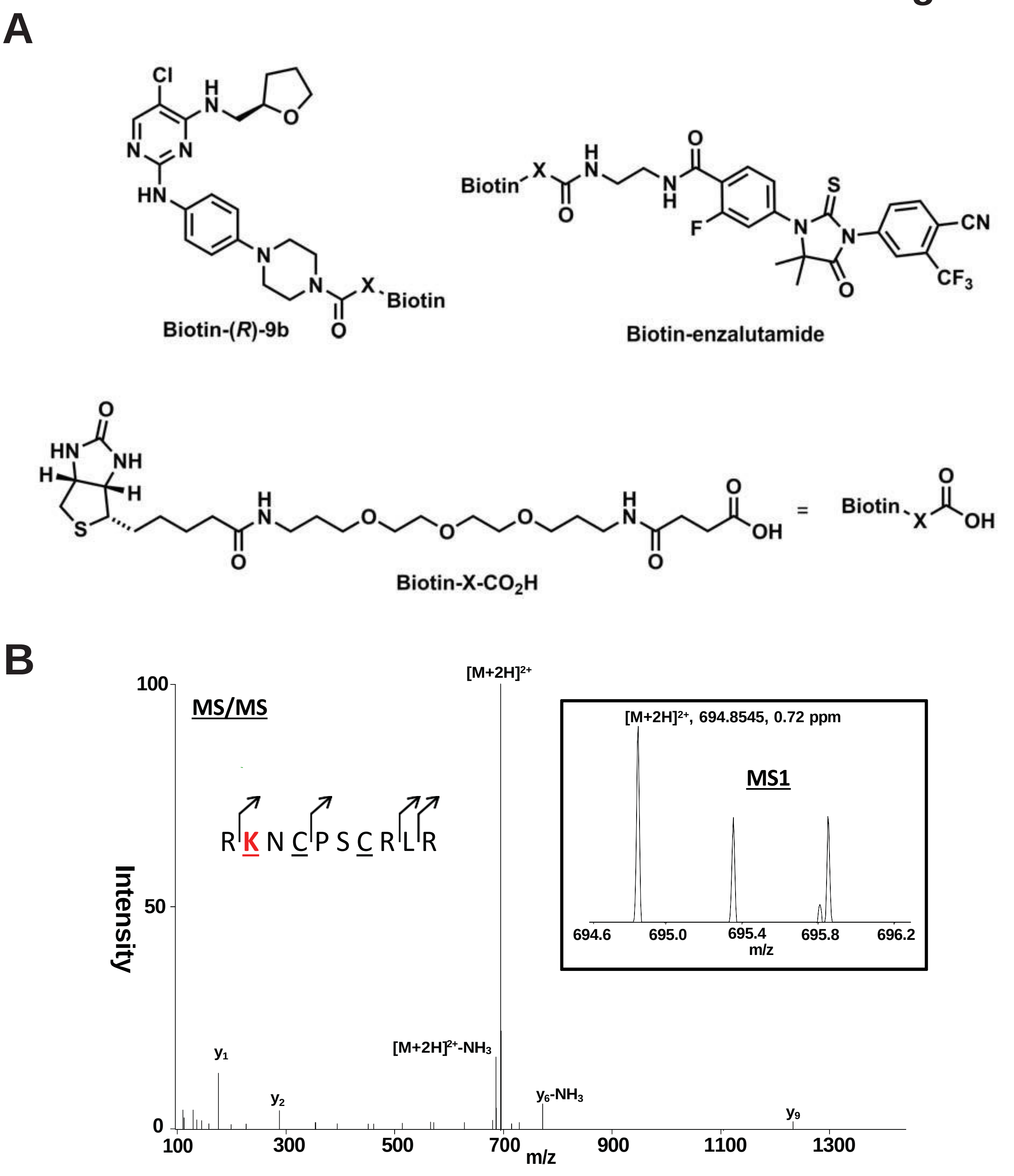

Figure S1. Detection of acK609-AR in Enzalutamide-resistant CRPC.
(A) Biotinylated Enzalutamide and (*R*)-**9b** were prepared. **(B)** The beads (at the final drug concentration of 25 nmol/50 µl beads) were incubated with nuclear lysates from enzalutamide resistant C4-2B cells. Bound proteins were eluted, electrophoresed, followed by mass spectrometry. AR peptide was observed as doubly charged, with an m/z value of 694.8545, a mass error of 0.72 ppm; The MS/MS spectrum was identified using Mascot with a score of 31.4.

Figure S2

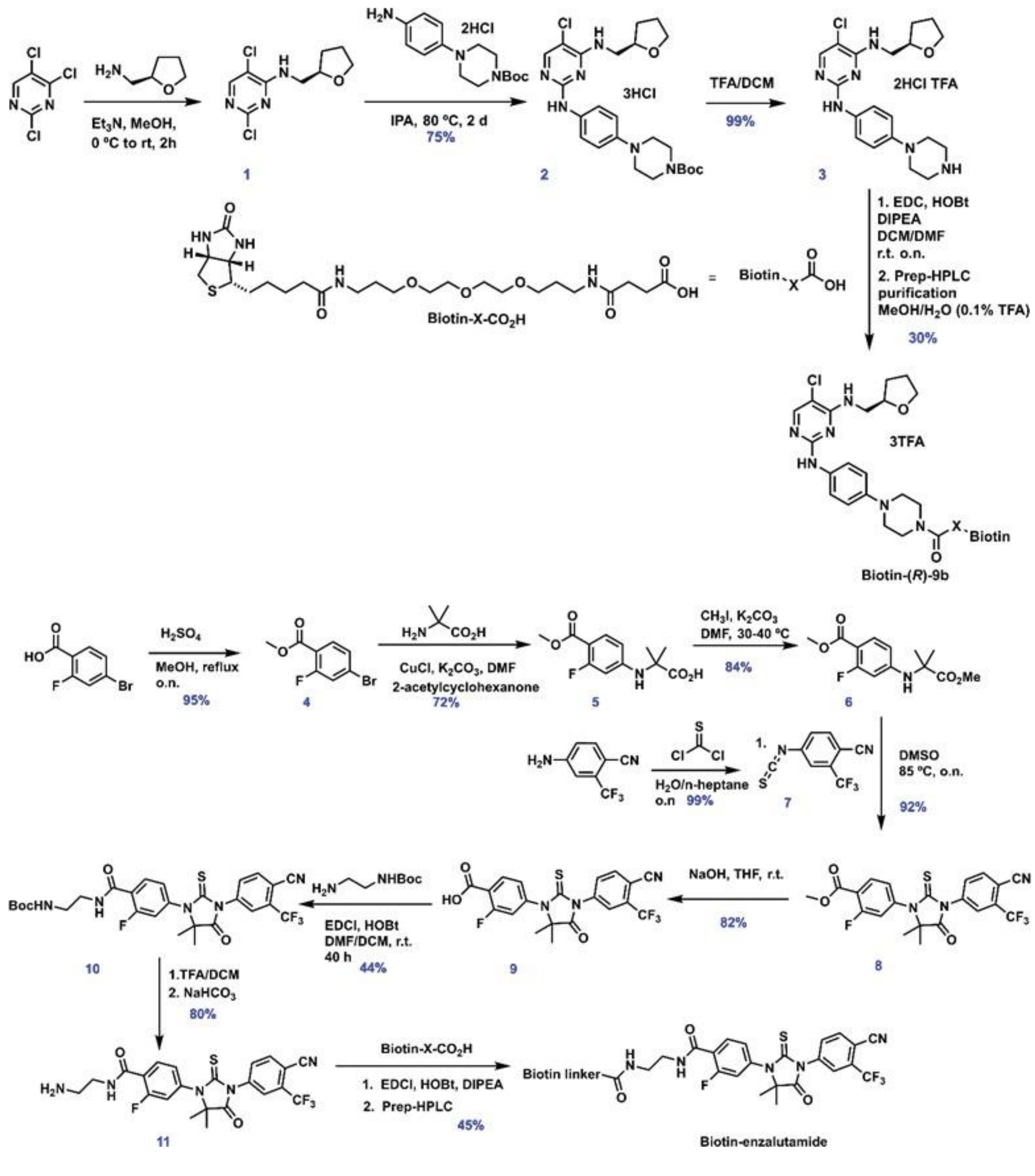

Figure S2. Linking enzalutamide and (*R*)-9b to biotin. Biotinylated **(*R*)-9b** was prepared by replacing its *N*-methyl group with the polyethylene-based linker conjugate *N*-(13-*N*-succinimidyl-4,7,10-trioxatridecanyl)biotinamide (biotin-X-CO_2H) (top panel). Similarly, biotinylated enzalutamide was prepared by attaching the biotin-X-CO_2H to an aminoethylene amide derivative of its *N*-methyl amide (bottom panel).

Figure S3

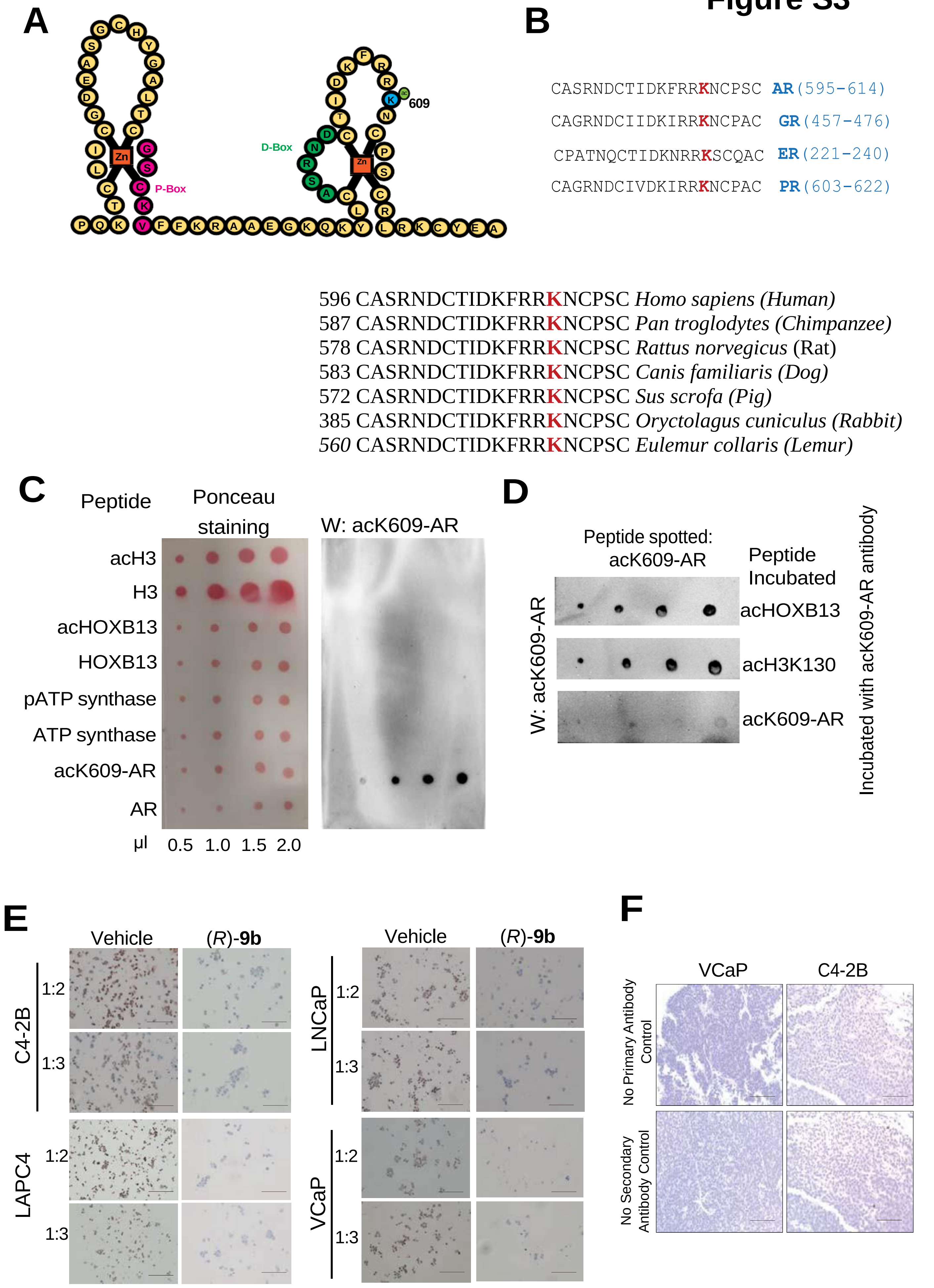
A
Zn
P-Box
D-Box
609
B
CASRNDCTIDKFRRRKNCPSC AR(595-614)
CAGRNDCIIDKIRRKNCPAC GR(457-476)
CPATNQCTIDKNRRKSCQAC ER(221-240)
CAGRNDCIVDKIRRKNCPAC PR(603-622)
596 CASRNDCTIDKFRRRKNCPSC Homo sapiens (Human)
587 CASRNDCTIDKFRRRKNCPSC Pan troglodytes (Chimpanzee)
578 CASRNDCTIDKFRRRKNCPSC Rattus norvegicus (Rat)
583 CASRNDCTIDKFRRRKNCPSC Canis familiaris (Dog)
572 CASRNDCTIDKFRRRKNCPSC Sus scrofa (Pig)
385 CASRNDCTIDKFRRRKNCPSC Oryctolagus cuniculus (Rabbit)
560 CASRNDCTIDKFRRRKNCPSC Eulemur collaris (Lemur)
C
Peptide
Ponceau staining
W: acK609-AR
acH3
H3
acHOXB13
HOXB13
pATP synthase
ATP synthase
acK609-AR
AR
µl 0.5 1.0 1.5 2.0
D
Peptide spotted: acK609-AR
Peptide Incubated
W: acK609-AR
acHOXB13
acH3K130
acK609-AR
Incubated with acK609-AR antibody
E
Vehicle
(R)-9b
C4-2B
1:2
1:3
LAPC4
LNCaP
VCaP
F
VCaP
C4-2B
No Primary Antibody Control
No Secondary Antibody Control

G

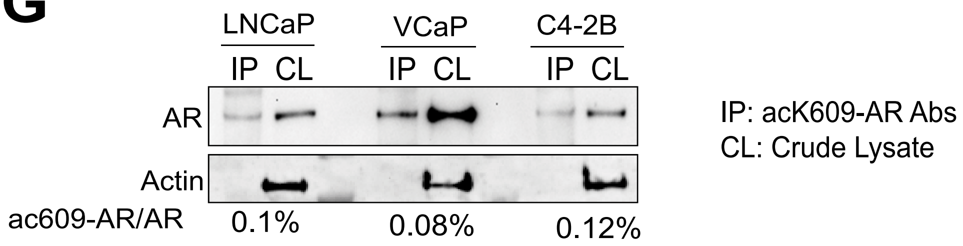

H

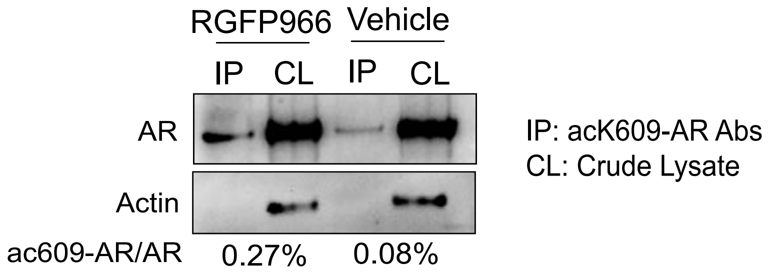

Figure S3. Conservation of K609 site and development of acK609-AR specific antibodies. **(A)** K609 residue is located on the second zinc finger of the AR DNA binding domain, distal to D-box element. (**B**) Alignment of nuclear receptor protein sequences revealed that K609 is conserved in AR, glucocorticoid receptor (GR), estrogen receptor (ER) ad progesterone receptor (PR). Further, K609 is fully conserved in the mammals that we checked (lower analysis). **(C)** The peptides corresponding to AR, acK609-AR, ATP synthase, pATP synthase, HOXB13, acHOXB13, H3 and acH3 were blotted onto Nitrocellulose membrane in a concentration dependent manner. The loaded peptides were visualized by Ponceau staining and were subjected to immunoblotting using acK609-AR antibody. **(D)** acK609-AR antibody was incubated with peptides for acK609-AR, acH3 and acHOXB13 respectively for 1h at RT, followed by incubation with membranes spotted with acK609-AR peptide and developed by western blot. **(E)** Vehicle or (*R*)-**9b** treated C4-2B, LAPC4, LNCaP and VCaP cells were fixed, pelleted using HistoGel and IHC stained using acK609-AR antibody. **(F)** IHC images of no primary and secondary antibody control are shown. **(G)** Enzalutamide treated LNCaP, VCaP and C4-2B cells were subjected to IP with acK609-AR antibody, followed by immunoblotting with AR antibodies. Crude lysates (CL) were also electrophoresed and the ratio of acK609-AR to total AR were measured by densitometry following western blotting. **(H)** C4-2B cells were treated with RGFP966 or vehicle and subjected to IP with acK609-AR antibody, followed by immunoblotting with AR antibodies. Crude lysates (CL) were also electrophoresed and the ratio of acK609-AR to total AR were measured by densitometry following western blotting.

Figure S4

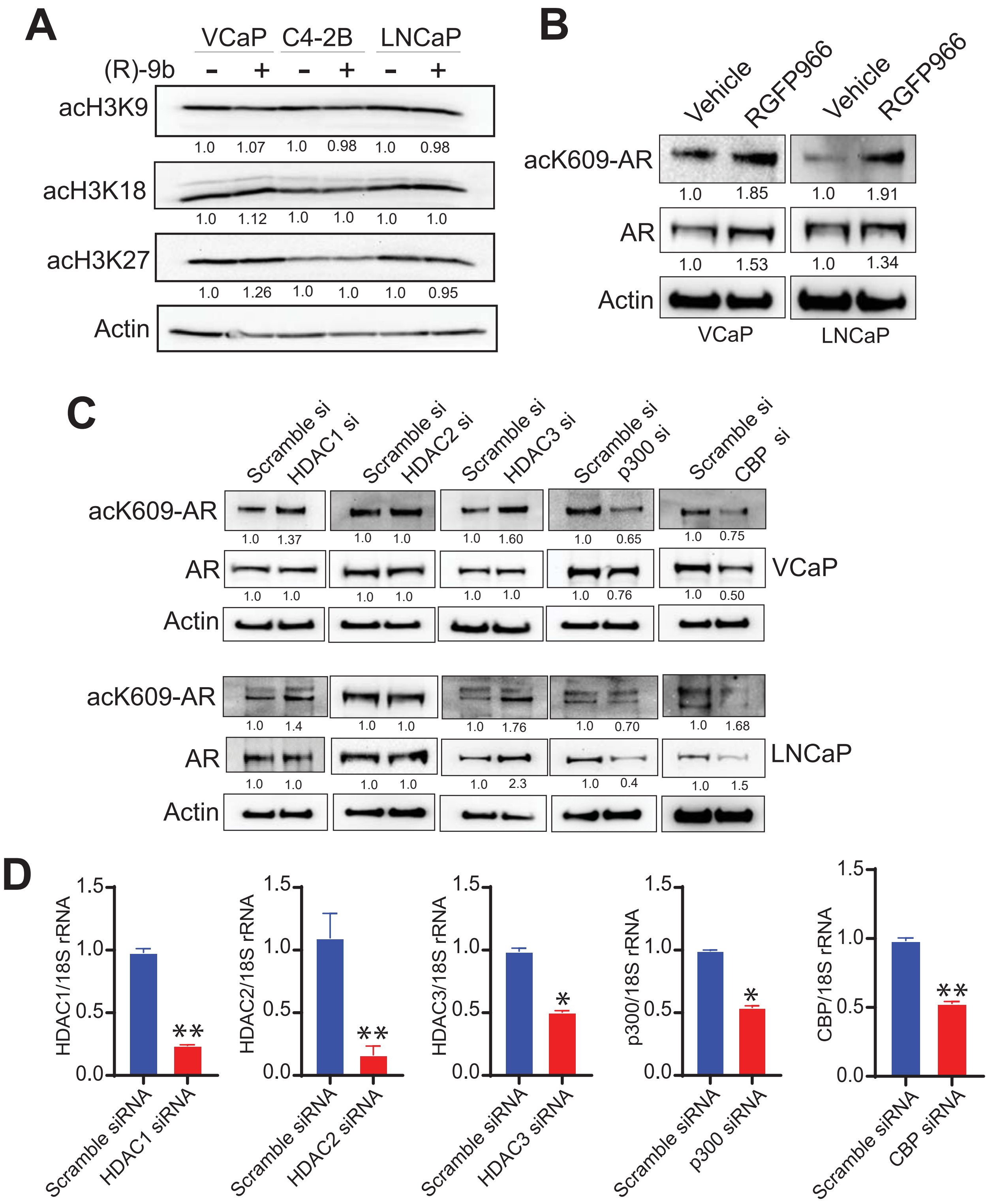

Figure S4. p300/CBP complex deposits acetylation marks on K609-AR, whereas HDAC1 and 3 are the histone deacetylases responsible for removing them.
(A) The effect of (R)-9b on global histone acetylation were studied by treating VCaP, C4-2B and LNCaP cells with (R)-9b and immunoblotted for acH3K9, acH3K18 and acH3K27 levels. **(B)** VCaP and LNCaP cells were treated with HDAC3 inhibitor RGFP966 and the lysates were immunoprecipitated for ac609-AR followed by immunoblotting with AR antibody. **(C)** VCaP and LNCaP cells were transfected with either scramble siRNA or siRNAs corresponding to HDAC1, HDAC2, HDAC3, p300 and CBP, followed by immunoprecipitation using acK609-AR antibody and western blot by AR antibody. **(D)** QPCR validating silencing of HDAC1, 2, 3, p300 and CBP following transfection with respective siRNAs in VCaP cells. Data respresented as +/- SEM.

Figure S5

A

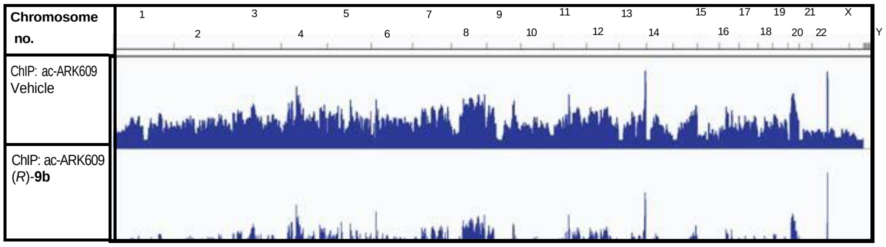

B

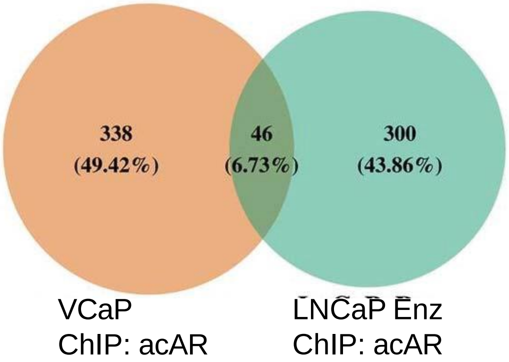

C

Homer Known Motif Enrichment Results (VCaP_acK609-AR)

Total Target Sequences = 727, Total Background Sequences = 41659

Rank	Motif	Name	P-value	log P-pvalue	q-value (Benjamini)	# Target Sequences with Motif	% of Targets Sequences with Motif	# Background Sequences with Motif
	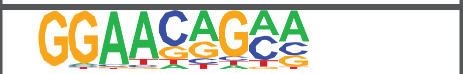	ZNF189(Zf)/HEK293-ZNF189.GFP-ChIP-Seq(GSE58341)/Homer	1e-14	-3.235e+01	0.0000	86.0	11.83%	1955.5
2	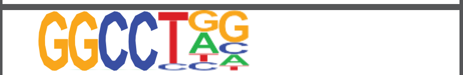	ZFX(Zf)/mES-Zfx-ChIP-Seq(GSE11431)/Homer	1e-7	-1.617e+01	0.0000	38.0	5.23%	819.4
3	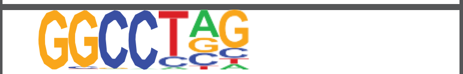	ZNF711(Zf)/SHSY5Y-ZNF711-ChIP-Seq(GSE20673)/Homer	1e-5	-1.260e+01	0.0005	41.0	5.64%	1066.5
4	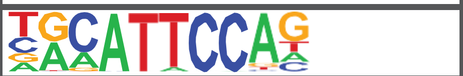	TEAD3(TEA)/HepG2-TEAD3-ChIP-Seq(Encode)/Homer	1e-5	-1.247e+01	0.0005	139.0	19.12%	5485.3
5	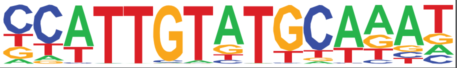	Oct4:Sox17(POU,Homeobox,HMG)/F9-Sox17-ChIP-Seq(GSE44553)/Homer	1e-3	-8.321e+00	0.0208	40.0	5.50%	1253.6
6	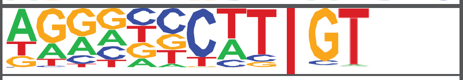	Sox9(HMG)/Limb-SOX9-ChIP-Seq(GSE73225)/Homer	1e-3	-8.235e+00	0.0208	68.0	9.35%	2512.7
7	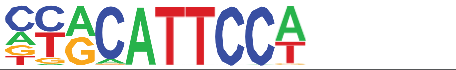	TEAD1(TEAD)/HepG2-TEAD1-ChIP-Seq(Encode)/Homer	1e-3	-7.327e+00	0.0402	108.0	14.86%	4554.2
8	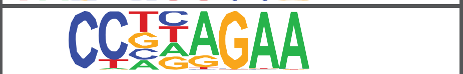	STAT6(Stat)/Macrophage-Stat6-ChIP-Seq(GSE38377)/Homer	1e-3	-7.266e+00	0.0402	81.0	11.14%	3229.9
9	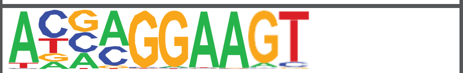	ELF5(ETS)/T47D-ELF5-ChIP-Seq(GSE30407)/Homer	1e-2	-5.853e+00	0.1366	63.0	8.67%	2517.4
10	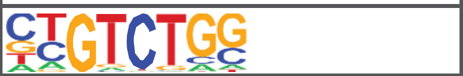	Smad2(MAD)/ES-SMAD2-ChIP-Seq(GSE29422)/Homer	1e-2	-5.438e+00	0.1860	49.0	6.74%	1891.2
11	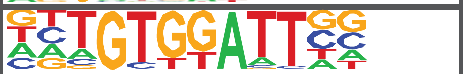	Foxh1(Forkhead)/hESC-FOXH1-ChIP-Seq(GSE29422)/Homer	1e-2	-4.953e+00	0.2748	86.0	11.83%	3780.3
12	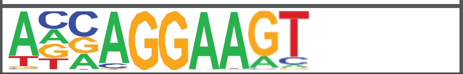	ELF3(ETS)/PDAC-ELF3-ChIP-Seq(GSE64557)/Homer	1e-2	-4.857e+00	0.2773	65.0	8.94%	2737.0

Figure S5. Genome-wide binding of acK609-AR in CRPCs and de novo motif enrichment. (A) acK609-AR ChIP-sequencing peak distribution in enzalutamide-resistant VCaP cells, in absence and presence of (*R*)-**9b**. (**B**) Venn diagrams summarizing the overlap between sites bound by acK609-AR in enzalutamide-resistant VCaP and enzalutamide-treated LNCaP cells. (**C**) acK609-AR ChIP-sequencing data was searched for the de novo transcription-factor-binding motif (analysis was performed using HOMER). Significantly enriched motifs and associated P values are shown.

Figure S6

Homer Known Motif Enrichment Result (LNCaP_Veh_motif)

Total Target Sequences = 653, Total Background Sequences = 46427

Rank	Motif	Name	P-value	log P-pvalue	q-value (Benjamini)	# Target Sequences with Motif	% of Targets Sequences with Motif	# Background Sequences with Motif	% of Background Sequences with Motif	Motif File	SVG
1	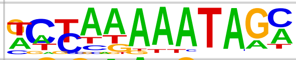	Mef2c(MADS)/GM12878-Mef2c-ChIP-Seq(GSE32465)/Homer	1e-6	-1.468e+01	0.0002	101.0	15.47%	4336.3	9.34%	motif file (matrix)	svg
2	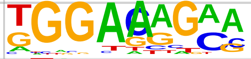	ZNF189(Zf)/HEK293-ZNF189.GFP-ChIP-Seq(GSE58341)/Homer	1e-5	-1.336e+01	0.0003	83.0	12.71%	3452.5	7.43%	motif file (matrix)	svg
3	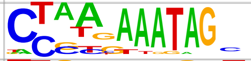	Mef2a(MADS)/HL1-Mef2a.biotin-ChIP-Seq(GSE21529)/Homer	1e-5	-1.152e+01	0.0014	90.0	13.78%	4027.2	8.67%	motif file (matrix)	svg
4	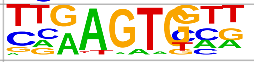	Bapx1(Homeobox)/VertebralCol-Bapx1-ChIP-Seq(GSE36672)/Homer	1e-4	-1.063e+01	0.0026	245.0	37.52%	13939.6	30.01%	motif file (matrix)	svg
5	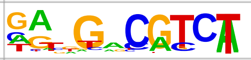	Atf1(bZIP)/K562-ATF1-ChIP-Seq(GSE31477)/Homer	1e-4	-1.061e+01	0.0026	88.0	13.48%	4010.5	8.63%	motif file (matrix)	svg
6	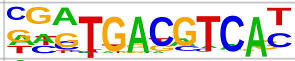	Atf7(bZIP)/3T3L1-Atf7-ChIP-Seq(GSE56872)/Homer	1e-4	-1.005e+01	0.0031	68.0	10.41%	2928.2	6.30%	motif file (matrix)	svg
7	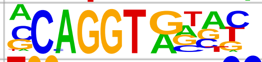	ZEB1(Zf)/PDAC-ZEB1-ChIP-Seq(GSE64557)/Homer	1e-3	-8.127e+00	0.0181	165.0	25.27%	9144.6	19.69%	motif file (matrix)	svg
8	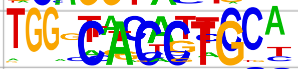	PRDM10(Zf)/HEK293-PRDM10.eGFP-ChIP-Seq(Encode)/Homer	1e-3	-8.094e+00	0.0181	68.0	10.41%	3135.6	6.75%	motif file (matrix)	svg
9	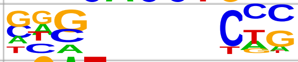	Slug(Zf)/Mesoderm-Snai2-ChIP-Seq(GSE61475)/Homer	1e-3	-7.532e+00	0.0255	67.0	10.26%	3143.5	6.77%	motif file (matrix)	svg
10	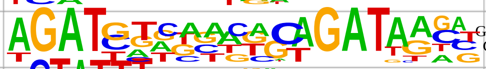	GATA3(Zf),DR8/iTreg-Gata3-ChIP-Seq(GSE20898)/Homer	1e-3	-6.930e+00	0.0419	13.0	1.99%	329.2	0.71%	motif file (matrix)	svg
11	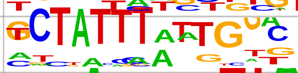	Mef2b.V5-ChIP-Seq(GSE67450) Mef2b(MADS)/HEK293-/Homer	1e-2	-6.795e+00	0.0436	113.0	17.30%	6061.9	13.05%	motif file (matrix)	svg
12	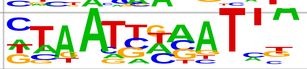	PROP1.biotin-ChIP- Prop1(Homeobox)/GHFT1- Seq(GSE77302)/Homer	1e-2	-6.617e+00	0.0477	73.0	11 8%	3618.4	7.79%	motif file (matrix)	svg
13	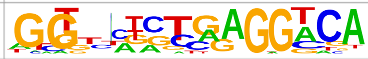	THRa(NR)/C17.2-THRa-ChIP-Seq(GSE38347)/Homer	1e-2	-6.099e+00	0.0739	62.0	9.49%	3030.3	6.52%	motif file (matrix)	svg
14	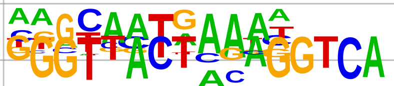	Hoxd12(Homeobox)/ChickenMSG-Hoxd12.Flag-ChIP-Seq(GSE86088)/Homer	1e-2	-5.659e+00	0.1066	196.0	30.02%	11739.9	25.28%	motif file (matrix)	svg
15	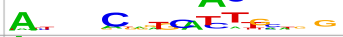	LXRE(NR),DR4/RAW-LXRb.biotin-ChIP-Seq(GSE21512)/Homer	1e-2	-5.005e+00	0.1913	28.0	4.29%	1192.8	2.57%	motif file (matrix)	svg
16	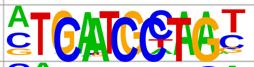	Atf4(bZIP)/MEF-Atf4-ChIP-Seq(GSE35681)/Homer	1e-2	-4.976e+00	0.1913	32.0	4.90%	1418.8	3.05%	motif file (matrix)	svg
17	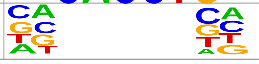	E2A(bHLH),near_PU.1/Bcell-PU.1-ChIP-Seq(GSE21512)/Homer	1e-2	-4.752e+00	0.2173	135.0	20.67%	7905.6	17.02%	motif file (matrix)	svg

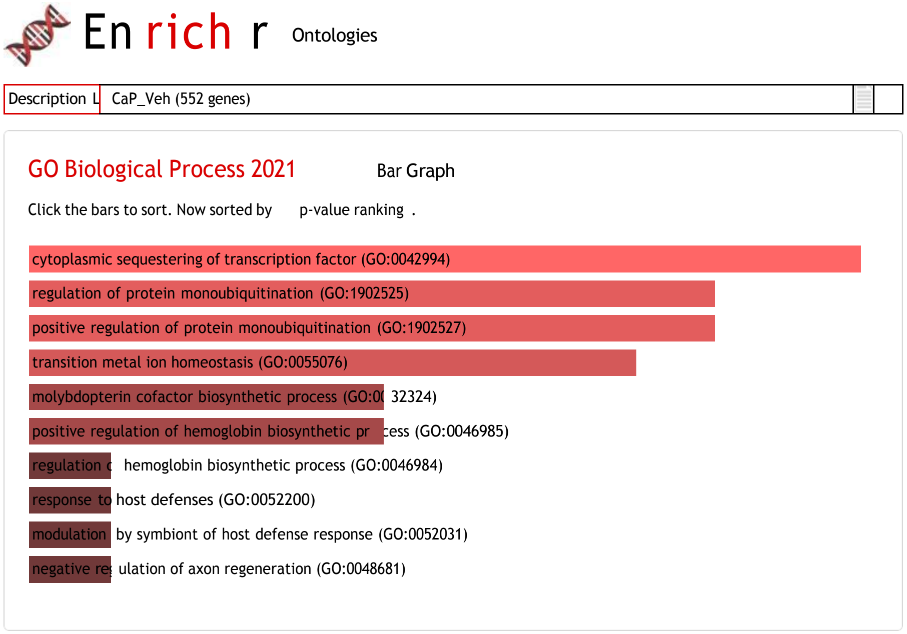

Figure S6. The de novo motif enrichment of acK609-AR in LNCaP cells and EnrichR analysis of biological processes.

(**Top**) acK609-AR ChIP-sequencing data was searched for the de novo transcription-factor-binding motif (analysis was performed using HOMER). The significantly enriched motifs and associated P values are shown. (**Bottom**) EnrichR analysis of biological processes regulated by acK609-AR in LNCaP cells.

Figure S7

Homer Known Motif Enrichment Result (LNCaP_Enz-Res_motif)

Total Target Sequences = 594, Total Background Sequences = 42580

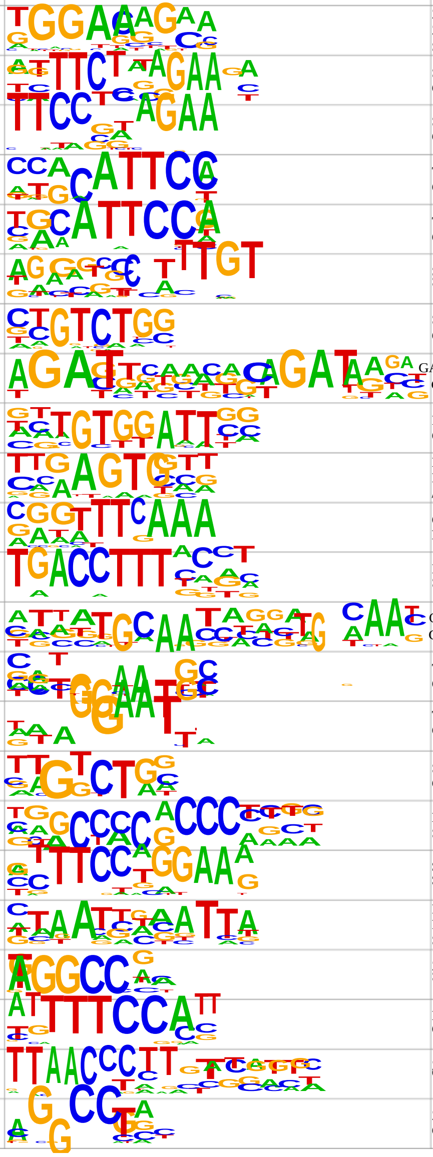

Rank	Motif	Name	P-value	log P-pvalue	q-value (Benjamini)	# Target Sequences with Motif	% of Targets Sequences with Motif	# Background Sequences with Motif	% of Background Sequences with Motif	Motif File	SVG
1		ZNF189(Zf)/HEK293-ZNF189.GFP-ChIP-Seq(GSE58341)/Homer	1e-25	-5.890e+01	0.0000	132.0	22.22%	3453.2	8.11%	motif file (matrix)	svg
2		STAT5(Stat)/mCD4+-Stat5-ChIP-Seq(GSE12346)/Homer	1e-19	-4.437e+01	0.0000	75.0	12.63%	1573.4	3.70%	motif file (matrix)	svg
3		STAT6(Stat)/Macrophage-Stat6-ChIP-Seq(GSE38377)/Homer	1e-9	-2.249e+01	0.0000	79.0	13.30%	2626.3	6.17%	motif file (matrix)	svg
4		TEAD1(TEAD)/HepG2-TEAD1-ChIP-Seq(Encode)/Homer	1e-9	-2.222e+01	0.0000	134.0	22.56%	5589.8	13.13%	motif file (matrix)	svg
5		TEAD3(TEA)/HepG2-TEAD3-ChIP-Seq(Encode)/Homer	1e-9	-2.079e+01	0.0000	149.0	25.08%	6587.1	15.47%	motif file (matrix)	svg
6		Sox9(HMG)/Limb-SOX9-ChIP-Seq(GSE73225)/Homer	1e-7	-1.692e+01	0.0000	80.0	13.47%	3037.9	7.13%	motif file (matrix)	svg
7		Smad2(MAD)/ES-SMAD2-ChIP-Seq(GSE29422)/Homer	1e-7	-1.679e+01	0.0000	115.0	19.36%	4988.6	11.72%	motif file (matrix)	svg
8		GATA3(Zf),DR8/iTreg-Gata3-ChIP-Seq(GSE20898)/Homer	1e-5	-1.357e+01	0.0001	19.0	3.20%	360.5	0.85%	motif file (matrix)	svg
9		Foxh1(Forkhead)/hESC-FOXH1-ChIP-Seq(GSE29422)/Homer	1e-5	-1.268e+01	0.0001	78.0	13.13%	3271.4	7.68%	motif file (matrix)	svg
10		Bapx1(Homeobox)/VertebralCol-Bapx1-ChIP-Seq(GSE36672)/Homer	1e-4	-1.130e+01	0.0005	200.0	33.67%	10989.5	25.81%	motif file (matrix)	svg
11		CHR(?)/Hela-CellCycle-Expression/Homer	1e-4	-1.079e+01	0.0008	72.0	12.12%	3111.1	7.31%	motif file (matrix)	svg
12		Nur77(NR)/K562-NR4A1-ChIP-Seq(GSE31363)/Homer	1e-4	-1.005e+01	0.0015	22.0	3.70%	590.6	1.39%	motif file (matrix)	svg
13		CEBP:CEBP(bZIP)/MEF-Chop-ChIP-Seq(GSE35681)/Homer	1e-4	-9.460e+00	0.0026	24.0	4.04%	704.8	1.66%	motif file (matrix)	svg
14		TEAD4(TEA)/Tropoblast-Tead4-ChIP-Seq(GSE37350)/Homer	1e-3	-9.144e+00	0.0033	92.0	15.49%	4465.3	10.49%	motif file (matrix)	svg
15		TEAD(TEA)/Fibroblast-PU.1-ChIP-Seq(Unpublished)/Homer	1e-3	-8.801e+00	0.0043	84.0	14.14%	4028.9	9.46%	motif file (matrix)	svg
16		Smad3(MAD)/NPC-Smad3-ChIP-Seq(GSE36673)/Homer	1e-3	-8.626e+00	0.0048	179.0	30.13%	10077.8	23.67%	motif file (matrix)	svg
17		KLF3(Zf)/MEF-Klf3-ChIP-Seq(GSE44748)/Homer	1e-3	-8.512e+00	0.0051	34.0	5.72%	1242.6	2.92%	motif file (matrix)	svg
18		STAT4(Stat)/CD4-Stat4-ChIP-Seq(GSE22104)/Homer	1e-3	-8.035e+00	0.0077	85.0	14.31%	4187.6	9.83%	motif file (matrix)	svg
19		Prop1(Homeobox)/GHFT1-PROP1.biotin-ChIP-Seq(GSE77302)/Homer	1e-3	-6.920e+00	0.0222	73.0	12.29%	3611.9	8.48%	motif file (matrix)	svg
20		ZFX(Zf)/mES-Zfx-ChIP-Seq(GSE11431)/Homer	1e-2	-6.504e+00	0.0321	73.0	12.29%	3669.2	8.62%	motif file (matrix)	svg
21		NFAT(RHD)/Jurkat-NFATC1-ChIP-Seq(Jolma_et_al.)/Homer	1e-2	-5.425e+00	0.0898	92.0	15.49%	5031.1	11.82%	motif file (matrix)	svg
22		ZNF652/HepG2-ZNF652.Flag-ChIP-Seq(Encode)/Homer	1e-2	-5.295e+00	0.0976	23.0	3.87%	905.2	2.13%	motif file (matrix)	svg
23		ZNF711(Zf)/SHSY5Y-ZNF711-ChIP-Seq(GSE20673)/Homer	1e-2	-4.814e+00	0.1511	96.0	16.16%	5407.7	12.70%	motif file (matrix)	svg

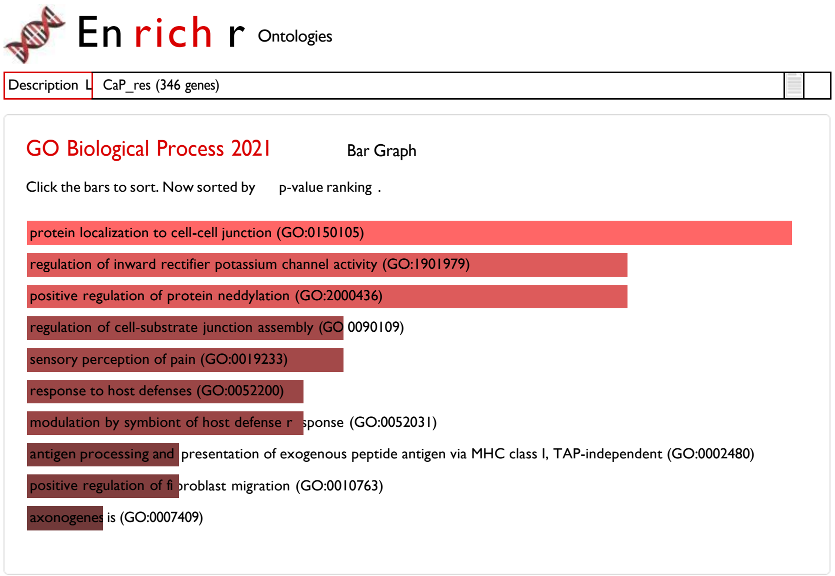

Figure S7. The de novo motif enrichment of acK609-AR in enzalutamide-treated LNCaP cells and EnrichR analysis of biological processes.

(**Top**) acK609-AR ChIP-sequencing data was searched for the de novo transcription-factor-binding motif (analysis was performed using HOMER). Ssignificantly enriched motifs and associated P values are shown. (**Bottom**) EnrichR analysis of biological processes regulated by acK609-AR in enzalutamide-treated LNCaP cells.

Figure S8

Homer Known Motif Enrichment Results (LNCaP_Res_vs_Veh_motif)

Rank	Motif	Name	P-value	log P-pvalue	q-value (Benjamini)	# Target Sequences with Motif	% of Targets Sequences with Motif	# Background Sequences with Motif	% of Background Sequences with Motif	Motif File	SVG
1	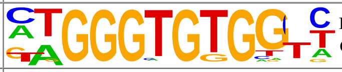	EKLF(Zf)/Erythrocyte-Klf1-ChIP-Seq(GSE20478)/Homer	1e-2	-6.826e+00	0.4644	13.0	4.39%	735.5	1.59%	motif file (matrix)	svg
2	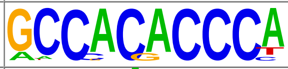	Klf4(Zf)/mES-Klf4-ChIP-Seq(GSE11431)/Homer	1e-2	-6.165e+00	0.4644	19.0	6.42%	1408.0	3.05%	motif file (matrix)	svg
3	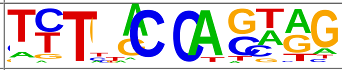	Bcl11a(Zf)/HSPC-BCL11A-ChIP-Seq(GSE104676)/Homer	1e-2	-5.584e+00	0.5361	37.0	12.50%	3636.4	7.88%	motif file (matrix)	svg
4	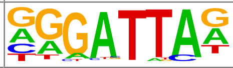	GSC(Homeobox)/FrogEmbryos-GSC-ChIP-Seq(DRA000576) /Homer	1e-2	-5.200e+00	0.5901	86.0	29.05%	10412.8	22.56%	motif file (matrix)	svg
5	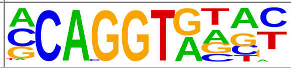	ZEB1(Zf)/PDAC-ZEB1-ChIP-Seq(GSE64557)/Homer	1e-2	-4.918e+00	0.6262	70.0	23.65%	8252.0	17.88%	motif file (matrix)	svg
6	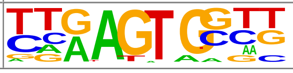	Bapx1(Homeobox)/VertebralCol-Bapx1-ChIP-Seq(GSE36672) /Homer	1e-2	-4.652e+00	0.6807	104.0	35.14%	13251.7	28.71%	motif file (matrix)	svg

Figure S8. The de novo motif enrichment of acK609-AR in vehicle versus enzalutamide-treated LNCaP cells.
The acK609-AR ChIP-sequencing data was searched for the de novo transcription-factor-binding motif (analysis was performed using HOMER). Significantly enriched motifs and associated P values are shown.

Figure S9

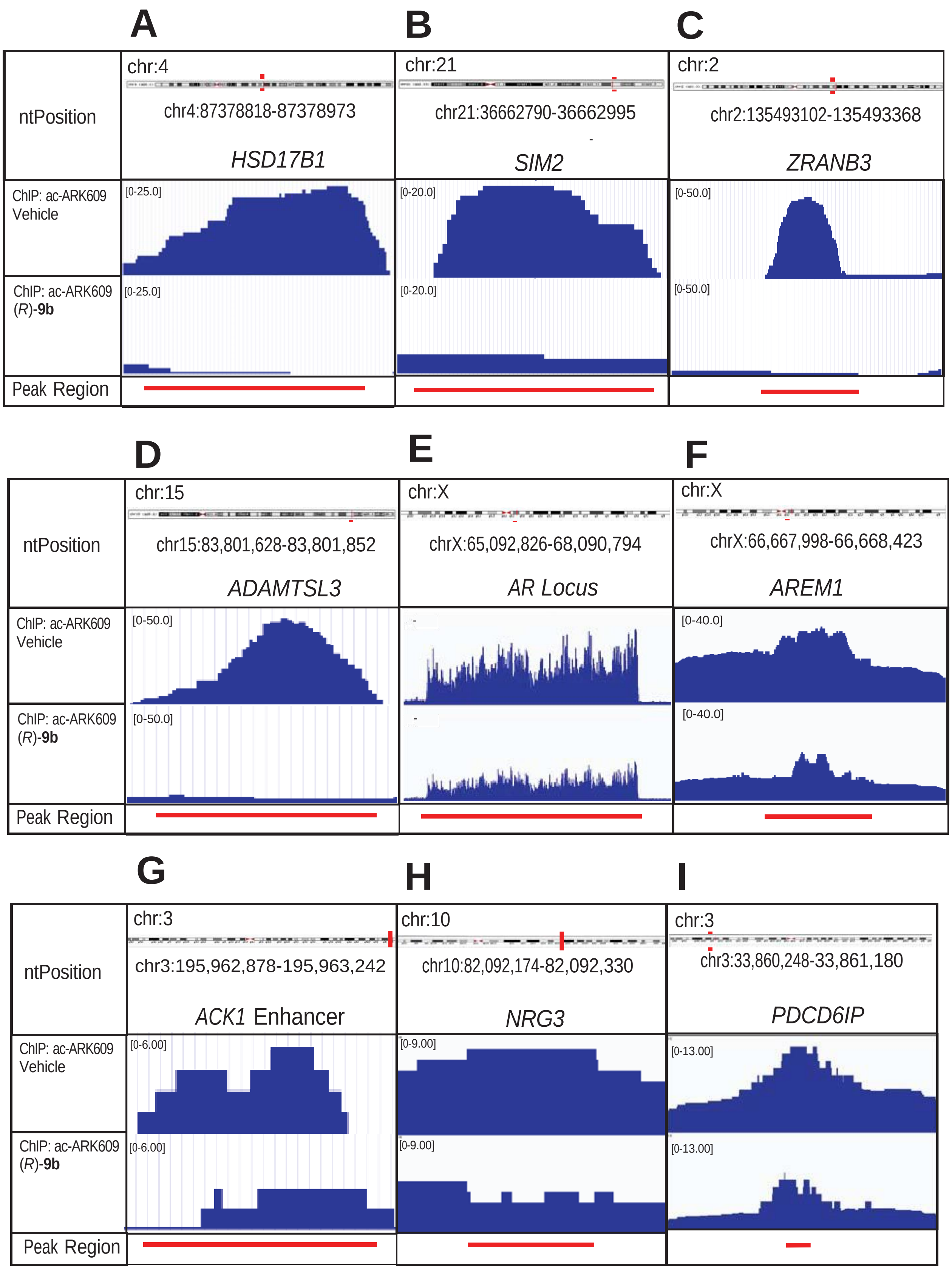

Figure S9. The acK609-AR ChIP-sequencing peaks in enzalutamide-resistant VCaP cells. (**A-I**) ChIP-sequencing peak distribution of acK609-AR in enzalutamide-resistant VCaP cells. ac-AR ChIP-sequencing peaks were searched for enriched sequence motifs for *HSD17B1* loci (A), *SIM2* loci (B), *ZRANB3* loci (C), *ADAMTSL3* loci (D), *AR* loci (E), AR enhancer (F), ACK1 enhancer (G), NRG3 (H), and PDCD6IP (I).

Figure S10

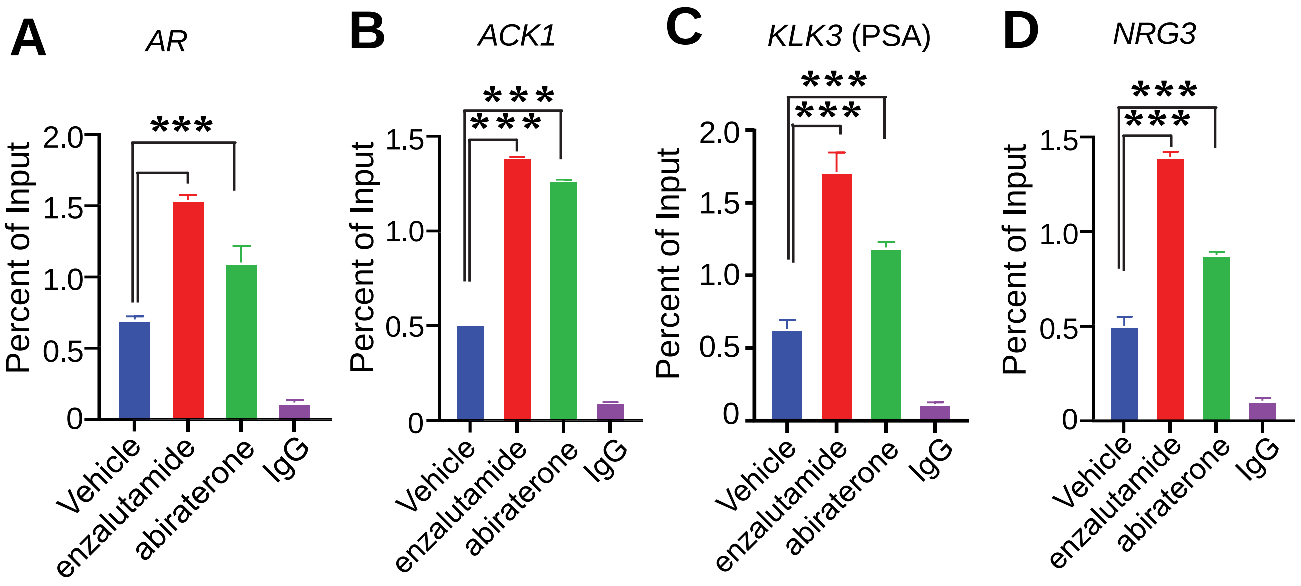

Figure S10. Resistant CRPC exhibits increased acK609-AR binding to target genes in presence of enzalutamide and abiraterone.

(**A-D**) ChIP was performed in enzalutamide- and abiraterone-treated (7μM, 7 days) C4-2B cells using acK609-AR antibody, followed by qPCR using primers corresponding to AR (**A**), ACK1 (**B**), PSA (**C**) and NRG3 (**D**) sites. For **A-D** (n=3, 3 replicates; a representative date is shown). For **F** and **G**, (n=2, 3 replicates; a representative data is shown). Data are represented as mean ± SEM. *p<0.05; ** p<0.01; ***p<0.001.

Figure S11

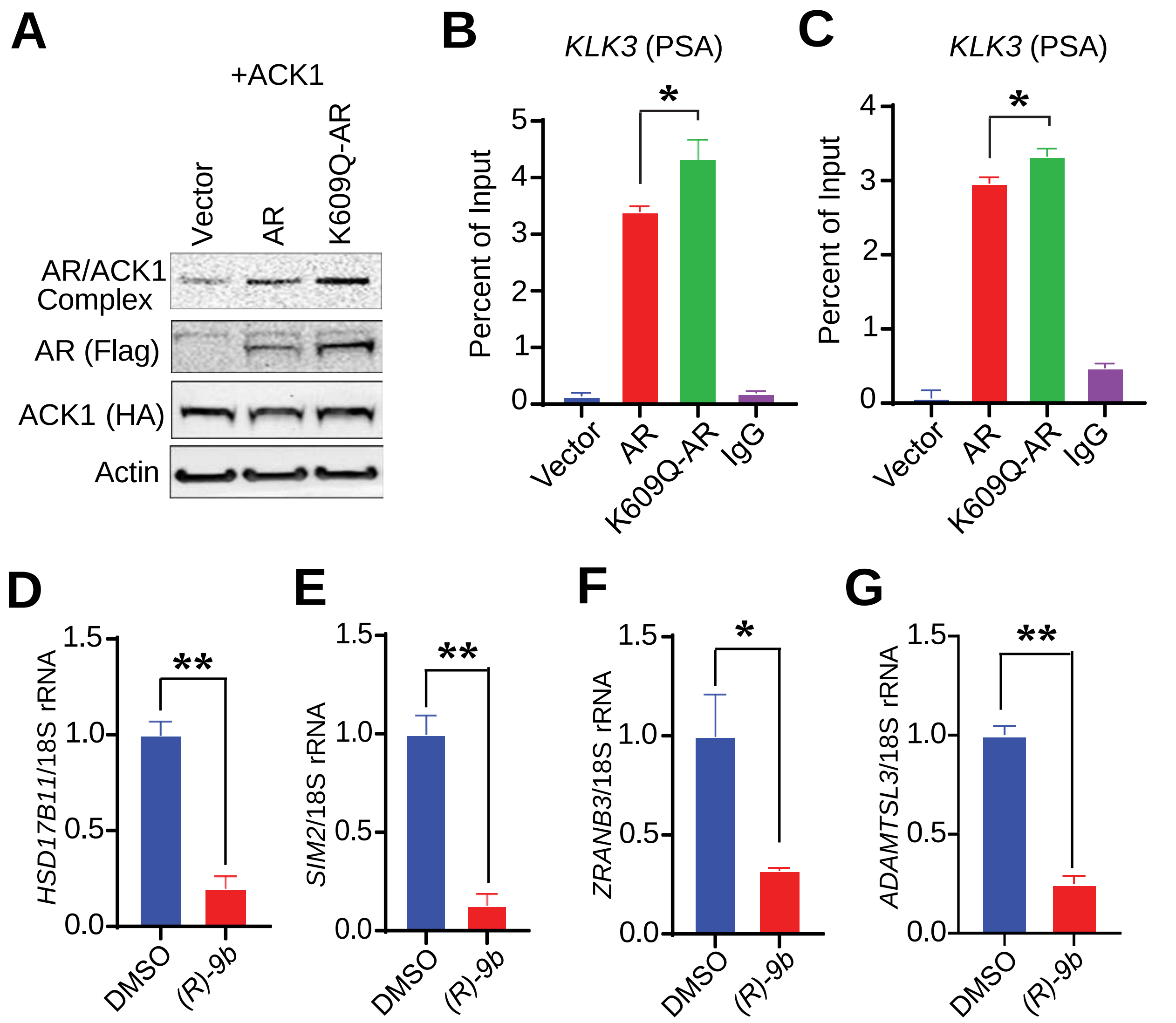

Figure S11. ACK1 inhibition using (*R*)-9b suppresses acK609-AR-regulated gene transcription.
Acetylation mimic K609Q-AR mutant exhibits enhanced chromatin binding. **(A)** HEK-293 cells were co-transfected with HA-tagged ACK1 with vector (pcDNA3.1), FLAG-tagged AR and K609Q-AR acetylation mimic mutant. 48h after transfection the lysates were subjected to co-IP with FLAG beads, followed by immunoblotting with HA antibody to detect AR/ACK1 complex (top panel). AR and ACK1 abundance were probed using FLAG and HA antibodies, respectively. **(B-C)** PC3 (**B**) and DU145 (**C**) cells were transfected with AR or K609Q-AR mutant and subjected to ChIP using FLAG antibody, followed by qPCR using primers corresponding to PSA ARE site. (**D-G**) VCAP cells were treated with DMSO or (*R*)-**9b**, RNA was extracted and subjected to QPCR using *HSD17B11*, *SIM2*, *ZRANB3* and *ADAMTSL3* primers. For **A-C** (n=2, 3 replicates; a representative date is shown). For **D** and **G**, (n=2, 3 replicates; a representative data is shown). Data are represented as mean ± SEM. *p<0.05; ** p<0.01; ***p<0.001.

Figure S12

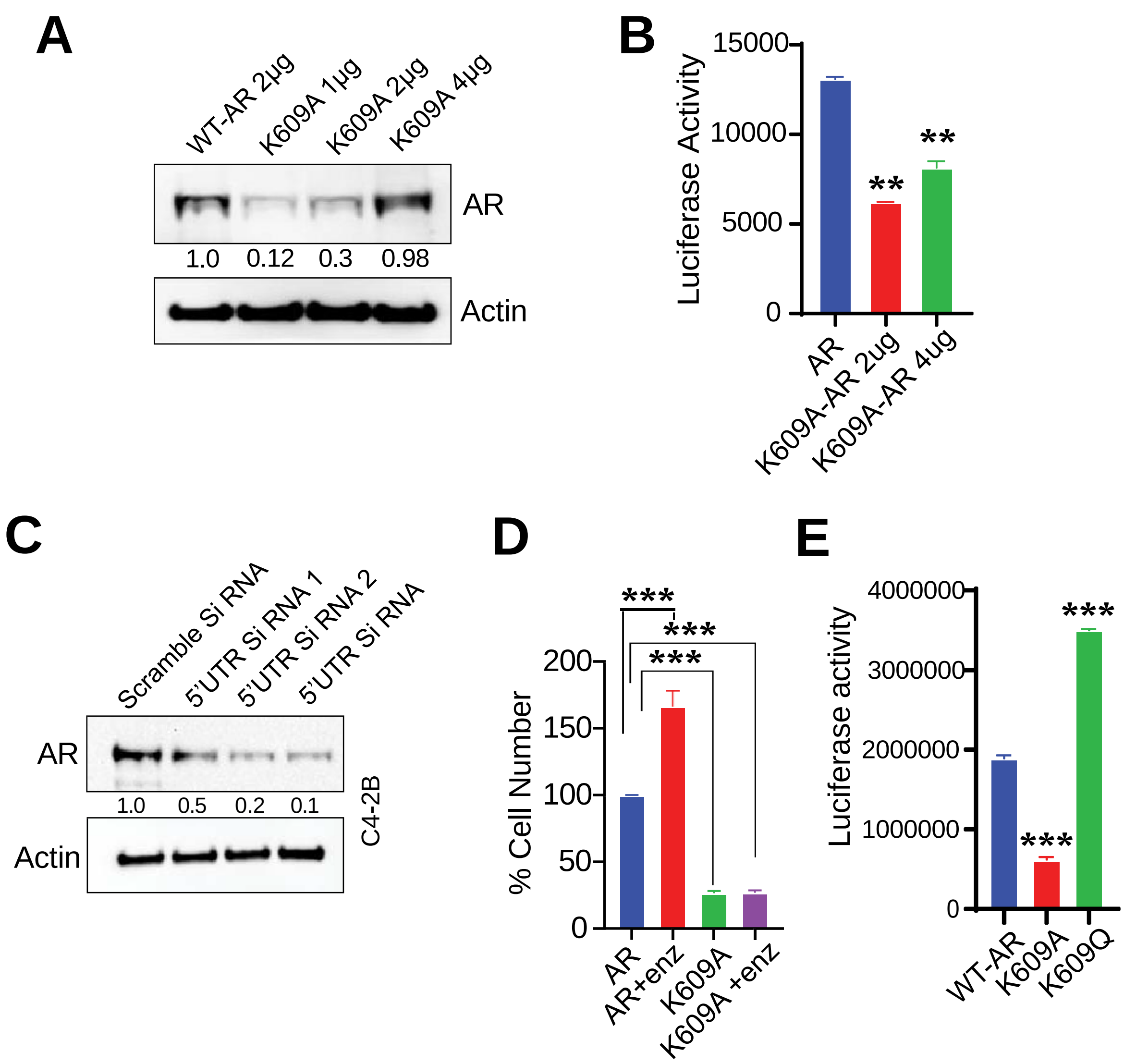

Figure S12. Acetylation at K609-AR confers resistance to enzalutamide by strengthening binding to ARE sites. (A) Concentration dependent expression of K609A-AR mutant plasmid in comparison to WT-AR in HEK293 cells. **(B)** HEK293 cells were transfected with AR or K609A-AR mutant and the ARR2PB-luciferase reporter construct. The cells were treated with 10 nM DHT overnight in serum-free media. Luciferase activity was determined 48 h after the transfection. **(C)** The endogenous AR expression in C4-2B and VCaP cells was silenced using AR 5' UTR specific oligos (1, 2 or pool) followed by Western blotting for total AR abundance. Actin was used as loading control. Densitometric analysis of the inhibition is shown below the AR blots. **(D)** C4-2B cells were transfected with AR 5'UTR specific siRNA followed by retro-viral infection using AR, K609A-AR mutant, or K609Q-AR mutant expressing viral particles. The cells were selected using puromycin, seeded in equal numbers and treated with enzalutamide for 72h followed by cell viability analysis using trypan blue. **(E)** Endogenous AR expression was silenced in LNCaP cells using AR 5'UTR specific siRNA followed by transfection using AR, K609A-AR mutant, or K609Q-AR mutant plasmids, along with the ARR2PB-luciferase reporter construct. The cells were treated with or without 10 nM DHT overnight in serum-free media. Luciferase activity was determined 72h after the transfection.

Figure S13

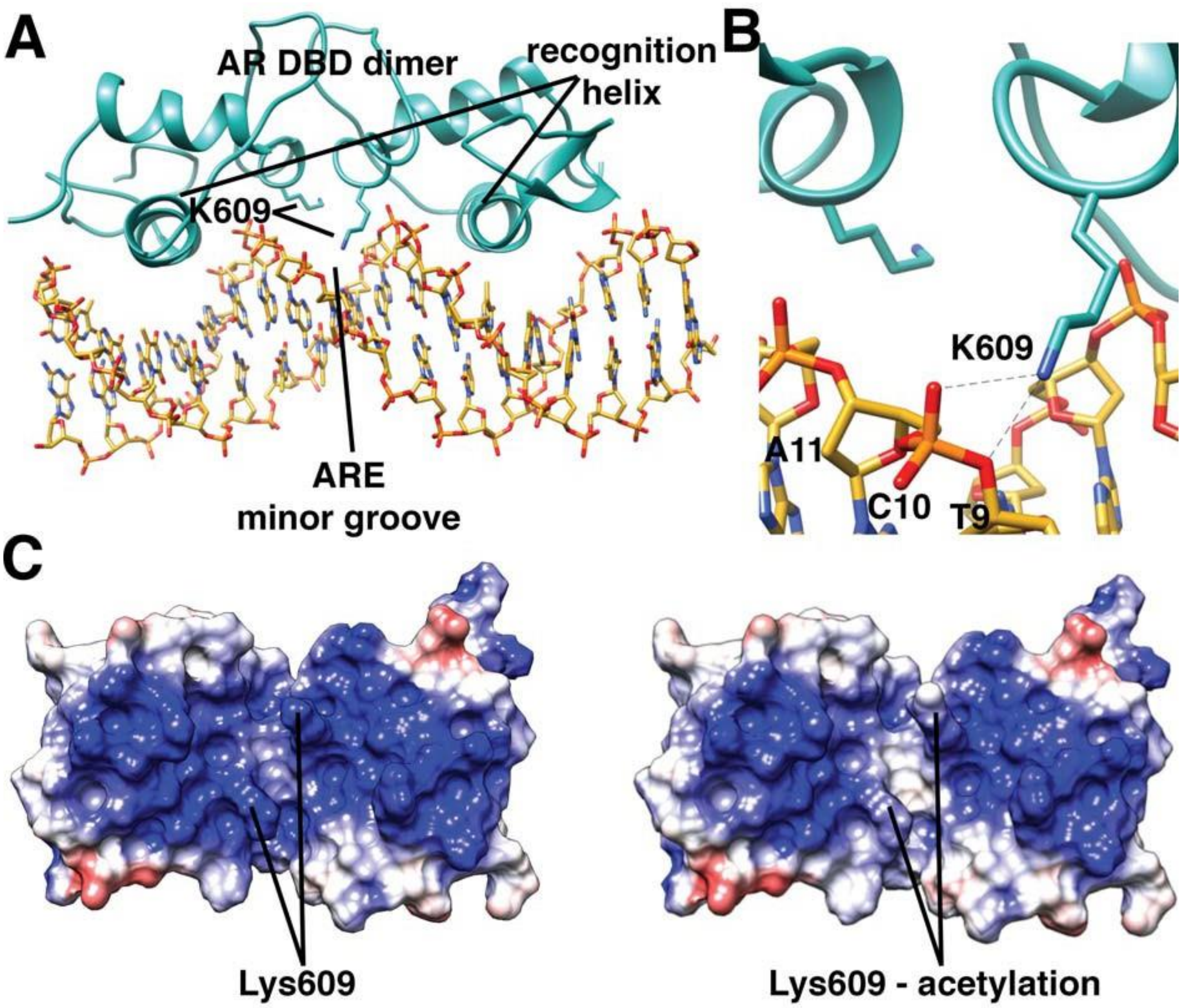

Figure S13. Model of the human androgen receptor (AR) DNA binding domain bound to the androgen response element (ARE).

(**A**) In the AR DNA binding domain dimer (cyan) the position of the ARE recognition helix in the major groove and Lys609 in the minor grove are indicated. Structure is based on the x-ray crystal structure of rat AR bound to the ARE. (**B**) Close-up of Lys609 interactions in the ARE minor groove. (**C**) Electrostatic surface comparison of the AR DNA binding domain containing Lys609 (left) and acetylated Lys609 (right). Electrostatic potential was calculated using Coulomb's law for deacetylated and acetylated lysine forms in Chimera. The potentials are on a red-white-blue [+10 to -10] color map in units of kJ/mol/e.

Figure S14

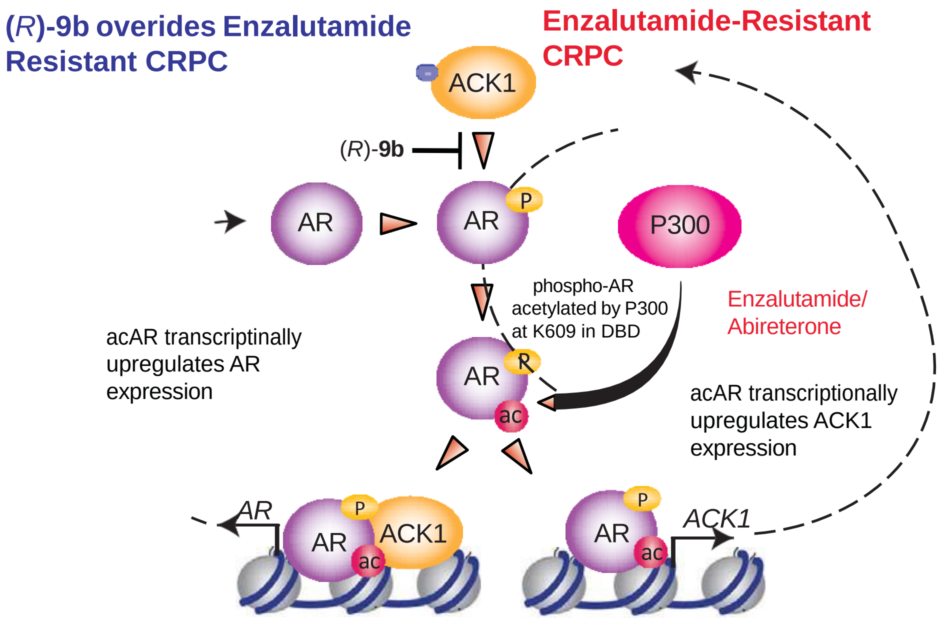

Figure S14. Dual-modified AR regulates AR and ACK1 expression to impart drug resistance in CRPCs (a model). ACK1 kinase phosphorylates AR, which is targeted by p300 for AR K609-acetylation in an enzalutamide-rich environment. The dual-modified AR binds *AR* and *ACK1* genetic loci to upregulate their expression, generating a feed-forward signaling loop that rapidly promotes drug resistance. Inhibition of AR phosphorylation and acetylation by (*R*)-**9b** not only lowered AR and ACK1, but also causedsensitization of enzalutamide-resistant CRPC tumor growth.

Table S1. The number of peaks obtained in acK609-AR and AR ChIP-Seq in VCaP and LNCaP cells. The total number of peaks are more than different peaks because of the duplication of certain gene-specific peaks.

	VCaP Vehicle ChIP: acK609-AR	VCaP (*R*)-9b ChIP: acK609-AR	VCaP Untreated ChIP: AR
3'UTR	**2**	**5**	**6**
5'UTR	**0**	**0**	**0**
Exon	**2**	**3**	**8**
Intergenic	**544**	**363**	**355**
Intron	**174**	**149**	**368**
Non-coding	**2**	**5**	**4**
Promoter-TSS	**2**	**7**	**10**
TTS	**1**	**1**	**9**
Total Peaks	**727**	**533**	**760**

	LNCaP Vehicle ChIP: acK609-AR	LNCaP-Enz ChIP: acK609-AR	LNCaP-Enz+(R)-9b ChIP: acK609-AR
3'UTR	**8**	**5**	**18**
5'UTR	**0**	**0**	**1**
Exon	**12**	**11**	**28**
Intergenic	**352**	**403**	**286**
Intron	**260**	**149**	**258**
Non-coding	**4**	**8**	**8**
Promoter-TSS	**10**	**8**	**25**
TTS	**7**	**10**	**14**
Total Peaks	**653**	**594**	**638**

Table S2. PCR primer sequences

**ChIP-PCR Primers**
AR CHIP FP4 (AREM1): TAGGAATCCAACTTACAAAG GAAGTGAAGG
AR CHIP RP4 (AREM1): GCAGTATGGCCATTTTCACG ATATCAATTA
ACK1 CHIP FP: CCCAGTGGCCGGGGA
ACK1 CHIP RP: AGAGCTGTGCCCAGTTTTATCT
NRG3 CHIP FP: CAGAACCTTCATGTGGCTGG
NRG3 CHIP RP: ACCACCCCACTACCCTTTTT
PDCD6IP CHIP FP: TGGATGTACTGTCTTTGAATCTCT
PDCD6IP CHIP RP: GGAAGAGCAATGCGAACTGA
PSA CHIP FP: CAACCCTGGACCTCACACCTA
PSA CHIP RP: GGAAATGACCAGGCCAAGAC
**qRT-PCR Primers:**
AR FP: ATGGTGAGCAGAGTGCCCTATC
AR RP: ATGGTCCCTGGCAGTCTCCAAA
ACK1 FP: ACTTTGGGCTGATGCGAGCACT
ACK1 RP: AAGGTGCGTGTCTTCAGGCTCT
ACTIN FP: CACCATTGGCAATGAGCGGTTC
ACTIN RP: AGGTCTTTGCGGATGTCCACGT
PSA FP: CGCAAGTTCACCCTCAGAAGGT
PSA RP: GACGTGATACCTTGAAGCACACC
TMPRSS2 FP: CAGGAGTGTACGGGAATGTG ATGGT
TMPRSS2 RP: GATTAGCCGTCTGCCCTCATT TGT
NRG3 FP: AGGACCTTGCATACTGTCTC
NRG3 RP: ACTCCTTGGTAGCCTTCTTT
PDCD6IP FP: GCTCAGATGAGAGAAGCCACCA
PDCD6IP RP: AGTCTGGATGCCTCCCTGTTCA
ADAMTSL3 FP: AGGCATCGTCTCACAAGTCCTG
ADAMTSL3 RP: CAGCCTTTGACACACCTGCTTTC
HSD17B11 FP: GACAGATGAACTGGCTGCCTTAC
HSD17B11 RP: CAGCCTGTTTACCACTTCCTCAG
ZRANB3 FP: GATGTTGCCGTAGCCTCAATGG
ZRANB3 RP: GGACACAGGCTCCATCTTCAAG
SIM2 FP: TGTCTTGGCGAAAAGGAACGCG
SIM2 RP: CCACAATCTGGTAGCAGGAGTC
18S rRNA FP: GGCCCTGTAATTGGAATGAGTC
18S rRNA RP:CCAAGATCCAACTACGAGCTT

Table S3. Sources of biochemicals, antibodies, cell lines, and software.

REAGENT or RESOURCE	SOURCE	IDENTIFIER
**Antibodies**		
AR (441)	Santa Cruz Biotech	Cat#sc-7305; RRID:AB_626671
ACK1	Santa Cruz Biotech	Cat#SC-28336; RRID: AB_626629
Phospho-tyrosine-HRP	Santa Cruz Biotech	Cat#SC-508; RRID: AB_628122
pACK1 (Tyr284)	Upstate	Cat#09-142; RRID: AB_612088
Actin Monoclonal Antibody	Sigma Aldrich	Cat#A5441; RRID: AB_476744
H4 Monoclonal Antibody	Cell Signalling	Cat#2935; RRID: AB_1147658
RNA Pol II	Active Motif	Cat#61667;RRID:AB_2687513
p300 Antibody	Santa Cruz Biotech	Cat# sc-81349, RRID:AB_1126965
IgG antibodies	Abcam	Cat#ab2410; RRID: AB_303052
Anti-Mouse secondary antibodies	Promega	Cat#W402B
SYBR Green RT-PCT Reagent kit	Applied Biosystem	Cat#4310179
SYBR PremixEx Taq II (Tli RNase H Plus)ROX	Clontech Takara	Cat#RR82LR
Anti-HA mouse monoclonal	Cell signaling	Cat#2367; RRID: AB_10691311
Anti-FLAG mouse monoclonal antibody	Sigma	Cat#F3165: RRID: AB_259529
**Bacterial and Virus Strains**		
DH5a subcloning efficiency	Invitrogen	Cat#18265017
**Biological Samples**		
Insulin	Sigma	Cat#10516-5ml
Fetal Bovine Serum	Life technologies	Cat#160044
Matrigel	Corning/Fisher	Cat#354428
**Chemicals**		
(R)-9b		N/A
Enzalutamide	Selleckchem	Cat#S1250
Abiraterone Acetate	Selleckchem	Cat#S2246
SBHA	Sigma	Cat# 559418
RGFP966	TargetMol	Cat# T6286
C646	Sigma	Cat#SML0002
DHT (Dihydrotestosterone)	Sigma	Cat#A8380
Trypan Blue	Sigma	Cat #T8154
DMSO (Dimethyl sulphoxide)	Sigma	Cat#D2650
EDTA	Sigma	Cat#E7889
DTT	Sigma	Cat#43815
HEPES	Sigma	Cat#H3537
Glycerol	Sigma	Cat#G9012
Streptavidin-sepharose beads	Cell Signaling	Cat#3419
Triton X-100	Sigma	Cat#T9284
Glycine	Sigma	Cat#G7126
Sodium chloride	Sigma	Cat#71386
Protease Inhibitor-EDTA free	Roche	Cat#11836170001
Protein-G magnetic beads	Bio-Rad	Cat#161-4023

Protein- A magnetic beads	Bio-Rad	Cat#161-4013
Insulin (from bovine pancreas)	Sigma	Cat#I5500
T3 (3’3’, 5-triiodo-L-thyronine)	Sigma	Cat#T2752
apo-Transferrin	Sigma	Cat#T4382
Protein A/G agarose beads	Santacruz	Cat# sc-2003
B-Mercaptoethanol	Sigma	Cat#M3148
Sodium OrthoVanadate	Fisher	Cat#S454-50
Sodium Fluoride	Sigma	Cat#S6521
**Critical Commercial Assays**		
ChIP-IT Express	Active motif	Cat#53008
RNeasy plus kit	Qiagen	Cat#74134
MinElute PCR purification kit	Qiagen	Cat#28004
HiSpeed Plasmid Maxi Kit	Qiagen	Cat#12663
Pierce ECL Western Blotting Kit	ThermoFisher	Cat#32106
Pierce ECL Plus Western Blotting Kit	ThermoFisher	Cat#32132
**Experimental Models: Cell Lines**		
LNCaP	ATCC	ATCC_ CRL-1740
VCaP	ATCC	ATCC_ CRL-2876
PC3	ATCC	ATCC_ CRL-1435
C4-2B	Dr. Evan Keller, Univ of Michigan	N/A
DU145	ATCC	ATCC_ HTB-81
**Experimental Models: Strains**		
Mice-SCID	Charles River	Strain code:236
**Software and Algorithms**		
GraphPad Prism Software		RRID:SCR_002798
**Other**		
DMEM low glucose	Gibco	Cat #11885-084
F12	Gibco	Cat#11765-054
RPMI	Gibco	Cat#11875-119
RPMI (No phenol)	Gibco	Cat#11835-0355
DMEM	Gibco	Cat#11965-092
IMDM	Gibco	Cat#12440-053
Trypsin-EDTA (0.05%)	Gibco	Cat#25300-054
1X DPBS	Gibco	Cat#14190-144

The following data files are available for download:

Data file S1. acK609-AR and AR ChIP-seq peaks in VCaP cells. (Excel file)

Data file S2. Protein-coding genes, ncRNAs, and pseudogenes in the Venn diagram in Figure 1F. (Word file)

Data file S3. acK609-AR ChIP-seq peaks in vehicle-treated LNCaP cells and enzalutamide-resistant LNCaP cells. (Excel)
